# CentromereArchitect: inference and analysis of the architecture of centromeres

**DOI:** 10.1093/bioinformatics/btab265

**Published:** 2021-07-12

**Authors:** Tatiana Dvorkina, Olga Kunyavskaya, Andrey V Bzikadze, Ivan Alexandrov, Pavel A Pevzner

**Affiliations:** Center for Algorithmic Biotechnology, Institute of Translational Biomedicine, Saint Petersburg State University, Saint Petersburg 199034, Russia; Center for Algorithmic Biotechnology, Institute of Translational Biomedicine, Saint Petersburg State University, Saint Petersburg 199034, Russia; Graduate Program in Bioinformatics and Systems Biology, University of California, San Diego, CA 92093, USA; Center for Algorithmic Biotechnology, Institute of Translational Biomedicine, Saint Petersburg State University, Saint Petersburg 199034, Russia; Department of Computer Science and Engineering, University of California, San Diego, CA 92093, USA

## Abstract

**Motivation:**

Recent advances in long-read sequencing technologies led to rapid progress in centromere assembly in the last year and, for the first time, opened a possibility to address the long-standing questions about the architecture and evolution of human centromeres. However, since these advances have not been yet accompanied by the development of the centromere-specific bioinformatics algorithms, even the fundamental questions (e.g. centromere annotation by deriving the complete set of human monomers and high-order repeats), let alone more complex questions (e.g. explaining how monomers and high-order repeats evolved) about human centromeres remain open. Moreover, even though there was a four-decade-long series of studies aimed at cataloging all human monomers and high-order repeats, the rigorous algorithmic definitions of these concepts are still lacking. Thus, the development of a centromere annotation tool is a prerequisite for follow-up personalized biomedical studies of centromeres across the human population and evolutionary studies of centromeres across various species.

**Results:**

We describe the CentromereArchitect, the first tool for the centromere annotation in a newly sequenced genome, apply it to the recently generated complete assembly of a human genome by the Telomere-to-Telomere consortium, generate the complete set of human monomers and high-order repeats for ‘live’ centromeres, and reveal a vast set of hybrid monomers that may represent the focal points of centromere evolution.

**Availability and implementation:**

CentromereArchitect is publicly available on https://github.com/ablab/stringdecomposer/tree/ismb2021

**Supplementary information:**

Supplementary data are available at *Bioinformatics* online.

## 1 Introduction

Since centromeric satellite repeats are among the longest and most difficult-to-assemble tandem repeats in the human genome, the problem of human centromere assembly was viewed as intractable until recently. As a result, most previous studies of associations between sequence variations and genetic diseases ignored ≈3% of the human genome. This is unfortunate since centromeres play crucial roles in chromosome segregation and a large component of genetic disease results from aneuploidies arising during meiosis (Nagaoka *et al.*, 2012). In addition, variations in centromeres are linked to cancer and infertility (Miga, 2019; Smurova and De Wulf, 2018; Zhu et al., 2018). Centromere sequencing is also important for addressing open problems about centromere evolution (Alkan *et al.*, 2007; Lower *et al.*, 2018; Shepelev *et al.*, 2009; Suzuki *et al.*, 2020) and the Centromere Paradox (Henikoff *et al.*, 2001), a surprising contrast between the highly conserved function and extremely fast evolution of centromeres. Other evolutionary puzzles are the broad range in centromere complexity, from simple point centromeres to long multi-megabase arrays (Malik and Henikoff, 2009), and the role of non-coding centromeric RNAs that are conserved across multiple species (Arunkumar and Melters, 2020). Moreover, the recent discovery of large archaic blocks of Neanderthal DNA spanning human centromeres reveals the potential of centromeres for studies of human population history (Langley *et al.*, 2019).

**Fig. 1.**
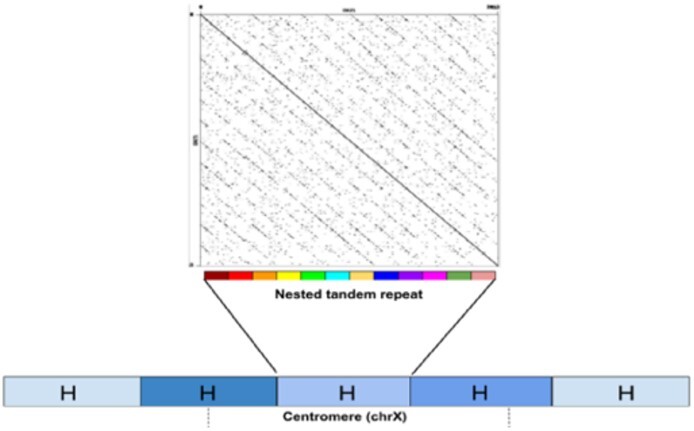
Architecture of centromere on chromosome X. The recently assembled centromere of chromosome X (cenX) consists of ∼18 000 monomers of length ≅171 bp each, as well a single copy of a LINE repeat based on the cenX assembly in Bzikadze and Pevzner (2020) [the latest T2T assembly (Nurk *et al*., 2021) represents a minor change to this assembly]. These monomers are organized into ∼1500 high-order repeats (HORs). Five HORs in the Figure are coloured by five shades of blue illustrating HOR variations. Each HORs is a nested tandem repeat formed by various monomers. The vast majority of HORs on cenX, referred to as *canonical HORs*, are formed by 12 monomers (shown by 12 different colors). Figure on top represents the dot plot of a canonical HOR that reveals 12 monomers. While HORs are 95–100% similar, monomers are only 65–88% similar. In addition to the canonical 12-monomer HORs, there is a small number of non-canonical HORs with varying numbers of monomers

*Alpha satellite arrays* in ‘live’ centromeres (that we refer to simply as centromeres) are extra-long tandem repeats that are formed by units repeating thousands of times with extensive variations in copy numbers in the human population (Black and Giunta, 2018) and limited nucleotide-level variations. Each such unit (referred to as a *high-order repeat* or *HOR*) represents a tandem repeat formed by smaller repetitive building blocks (referred to as *monomers*), thus forming a *nested tandem repeat* (Fig. 1). Each human monomer is of length ≅171 bp and each HOR is formed by multiple monomers that differ from each other. For example, the vast majority of HORs units on the centromere of the human X chromosome (referred to as cenX) consist of 12 monomers. Although different HOR units on cenX are highly similar (95–100% sequence identity), the 12 monomers forming each HOR are rather diverged (65–88% sequence identity). In addition to standard 12-monomer HOR units, some units on cenX have a non-canonical monomer structure: 35 out of 1510 units are formed by a smaller or larger number of monomers than the canonical 12-monomer HOR (Bzikadze and Pevzner, 2020). The tandem repeat structure of human centromeres may be interrupted by retrotransposon insertions (for example, cenX has a single insertion of a LINE element).

Recent advances in long-read sequencing technologies and bioinformatics algorithms led to rapid progress in centromere assembly in the last year (Bzikadze and Pevzner, 2020; Miga *et al.*, 2020; Nurk *et al.*, 2020) and, for the first time, opened a possibility to address the long-standing questions about the architecture and evolution of human centromeres. Recent evolutionary studies of centromeres (Bzikadze and Pevzner, 2020; Suzuki *et al.*, 2020; Uralsky *et al.*, 2019) revealed the importance of partitioning them into monomers, the problem that was recently addressed by the StringDecomposer algorithm (Dvorkina *et al.*, 2020). StringDecomposer takes a monomer-set and a genomic segment and partitions this segment into (monomeric) *blocks* (each block is similar to one of the monomers). For each monomer *M*, it generates the set of *M-blocks* in the centromere (that are more similar to *M* than to other monomers) and translates the centromere from the nucleotide alphabet into the alphabet of monomers.

StringDecomposer opened a possibility to generate the complete set of human HORs, the problem that remains unsolved despite multiple studies in the last four decades (Alexandrov *et al.*, 2001; Alkan *et al.*, 2007; McNulty and Sullivan, 2018; Paar *et al.*, 2005; Sevim *et al.*, 2016; Shepelev *et al.*, 2015; Uralsky *et al.*, 2019; Waye and Willard, 1985). However, the challenge of properly defining the set of all human monomers remained outside the scope of the String Decomposition Problem. As a result, many questions about centromere architecture and evolution remain unanswered, e.g. it remains unclear how to define the complete set of human monomers (a prerequisite for launching StringDecomposer) and HORs, moreover, the rigorous algorithmic definitions of these concepts are still lacking. Since the Human Pangenome Reference Consortium (https://humanpangenome.org) aims to generate 100s of complete human genomes in 2021, there is an urgent need for a fully automated centromere annotation (monomer and HOR inference) in newly sequenced complete genomes. We developed the CentromereArchitect tool that addressed the monomer and HOR inference problems described below.

*Monomer inference problem.* Although Sevim *et al.* (2016) constructed a large set of human monomers, this set is still missing some monomers, particularly rare monomers. Moreover, Uralsky *et al.* (2019), Bzikadze and Pevzner (2020) and Dvorkina *et al.* (2020) have shown that the evolution of centromeres often results in still underexplored *hybrid monomers* (that represent a concatenate of a suffix of one known monomer with a prefix of another known monomer) and hypothesized that they represent the driving force for the ‘birth’ of new monomers. We describe the MonomerGenerator algorithm for identifying all monomers in the human genome and construct a comprehensive set of human monomers for live centromeres that includes many rare and hybrid monomers that evaded identification in previous studies.

*HOR inference problem.* Human centromeres are formed by complex HORs, that are in turn formed by chromosome-specific monomers. Although previous studies derived lists of the most abundant human HORs (McNulty and Sullivan, 2018; Shepelev *et al.*, 2015), there are still many HORs that remain to be discovered. Moreover, previous studies often derived HORs using heuristic/manual approaches and have not even defined a rigorous computational concept of a HOR. Below we define the concept of a HOR and reveal that HORs are organized into even more complex repeat structures that we refer to as *superHORs*. We describe the HORDecomposer algorithm for inferring HORs and superHORs, infer them from live centromeres of the entire human genome, and reveal many previously unknown HORs. We further define the notion of a *HOR-graph* and show how a selection of a single HOR in each connected component of this graph (called a *primary HOR*) parallels decades of previous research (Alexandrov *et al.*, 2001; Alkan *et al.*, 2007; Paar *et al.*, 2005; Sevim *et al.*, 2016; Uralsky *et al.*, 2019).

## 2 Materials and methods

**Datasets**. We extracted the satellite arrays from the assembly (public release v1.0) of the haploid CHM13 cell line (https://github.com/nanopore-wgs-consortium/chm13#v10) constructed by the Telomere-to-Telomere (T2T) consortium (Logsdon *et al.*, 2021; Miga *et al.*, 2020; Nurk *et al.*, 2021). Supplementary Note ‘Information About Human Centromeres’ presents the coordinates of extracted regions for all live human centromere arrays.

**Monomer Inference Problem.** Given a string *Centromere* and a string-set *Monomers*, the StringDecomposer tool (Dvorkina *et al.*, 2020) decomposes the *Centromere* into (monomeric) *blocks* [we refer to the resulting block-set as *Blocks*(*Centromere, Monomers*)]. For each block *Block*, StringDecomposer assigns the value *div*_1_(*Block*) [*div*_2_(*Block*)] that represents the *divergence* between this block and its most similar monomer (its second-most similar monomer). The divergence between a pair of strings is defined as the edit distance between them divided by the length of the longest string.

Given a monomer *M* from the monomer-set *Monomers*, we refer to a block from *Blocks*(*Centromere, Monomers*) as an *M-block* if *M* is a most similar monomer to this block (ties are broken arbitrarily). The *M-consensus* is defined as the consensus of the multiple alignment of all *M*-blocks. Given monomers *M* and *M′*, we denote the edit distances between the *M*-consensus and the *M′*-consensus as *distance*(*M, M′*). The *separation* of a monomer *M* [referred to as *separation*(*M*)] is defined as the shortest distance between *M* and all other monomers. The *radius* of a monomer *M* [referred to as *radius*(*M*)] is defined as the maximum edit distance between its *M*-consensus and all *M*-blocks. *The separation ratio* of a monomer *M* is defined as *separationRatio*(*M*) = *separation*(*M*)/*radius*(*M*).

The *count* of a monomer *M* [referred to as *count*(*Centromere*, *M*)] is defined as the number of *M*-blocks in *Blocks*(*Centromere, Monomers*). A monomer is classified as *frequent* if its count exceeds the threshold |*Blocks*(*Centromere, Monomers*)|/*FreqCeiling* (default value *FreqCeiling =* 40), and *infrequent*, otherwise. An infrequent monomer is classified as *rare* if its count does not exceed a threshold *rareMonomerCount* (default value *rareMonomerCount* = 5).

We classify a block *Block* as *resolved* if *div*_1_(*Block*) is below the threshold *maxResolvedDivergence* (default value *maxResolvedDivergence* = 5%). We refer to the *Block* as *non-monomeric* if *div*_1_(*Block*) exceeds the threshold *maxDivergence* (default value *maxDivergence *=* *40%). Finally, a *Block* is *unresolved* if it is neither *resolved* nor *non-monomeric*.

We say that a monomer-set *Monomers resolves* a centromere *Centromere* if the fraction of resolved blocks in this centromere exceeds the threshold *FractionResolvedBlocks* and all other blocks are non-monomeric (default value *FractionResolvedBlocks* = 0.95). Given an integer *Length*, we say that a monomer-set is *Length*-uniform if all monomers in this set have a length similar to *Length*, i.e. that differs from *Length* by at most *MaxLengthDivergence*, where *MaxLengthDivergence* is a parameter (the default value is 0.01*Length).

**Monomer Inference Problem.**

**Input.** A string *Centromere* and parameters *maxResolvedDivergence, Length, MaxLengthDivergence* and *FractionResolvedBlocks.*

**Output.** A *Length*-uniform monomer-set *Monomers* that resolves *Centromere* and has a minimum number of monomers among all *Length*-uniform monomer-sets that resolve *Centromere.*

Previous attempts to generate monomers used a single consensus monomer *M* (e.g. a consensus of all human alpha satellites) to partition a centromere into *M*-blocks and further cluster these blocks using single-linkage clustering (Sevim *et al.*, 2016). Although this approach succeeded in deriving many human monomers, it does not necessarily resolve a centromere, particularly in the case of clusters that result in monomers with large radius. Below we describe a simple MonomerGenerator algorithm for an approximate solution of the Monomer Inference Problem.

**MonomerGenerator algorithm.** In addition to a string *Centromere*, MonomerGenerator has two input parameters: a threshold *maxResolvedDivergence*, and a string *InitialMonomer* (note the difference with the Monomer Inference Problem with respect to parameters). It is an iterative algorithm that gradually extends the monomer-set, starting with the monomer-set that consists of a single monomer *InitialMonomer.* In the case of the human genome, it sets *InitialMonomer = ConsensusMonomer*, where *ConsensusMonomer* is specified in Supplementary Note ‘Consensus monomer and reference monomers.’

**Fig. 2.**
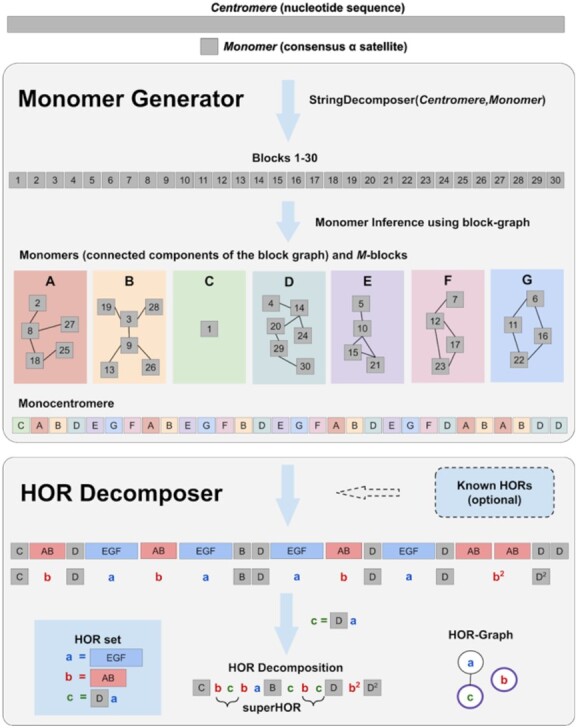
CentromereArchitect pipeline. As an Input, CentromereArchitect takes the nucleotide sequence *Centromere* of a centromere (or nucleotide sequences of all centromeres) and a consensus alpha satellite sequence *Monomer*. CentromereArchitect consists of two modules MonomerGenerator and HORDecomposer. MonomerGenerator constructs the block-graph and extracts each monomer from a connected component of this graph. It further uses the constructed monomer-set to partition the centromere into *M*-blocks using StringDecomposer. This partitioning transforms centromere into monocentromere. HORDecomposer infers the set of HORs and partitions the monocentromere into these HOR. As an optional input, HORDecomposer can incorporate known HORs in its Output. Finally, HORDecomposer infers superHORs in the resulting HOR decomposition and constructs the HOR-graph. Each connected component of that graph depicts a HOR-component and the vertex with the purple border corresponds to the primary HOR of that HOR-component

Given a string *Centromere* and a monomer-set *Monomers*, MonomerGenerator launches StringDecomposer to generate the block-set *Blocks*(*Centromere, Monomers*) and constructs the *block-graph* where vertices are unresolved blocks and edges connect unresolved blocks with divergence below *maxResolvedDivergence*/2 (Fig. 2). Since the block-set for the entire human genome contains nearly 300 000 blocks, the brute-force construction of the block-graph (that requires computing the edit distance between all pairs of blocks) faces the running time bottleneck. Supplementary Note ‘Constructing connected components of the block-graph’ describes a fast algorithm for constructing connected components of the block-graph.

MonomerGenerator selects a largest connected component (with a maximum number of vertices) in the constructed block-graph and computes its consensus *newMonomer* by constructing the multiple alignment of all blocks (vertices) in this component using Clustal Omega (Sievers *et al.*, 2011). Afterward, MonomerGenerator extends the monomer-set by adding *newMonomer* and iterates until the monomer-set resolves *Centromere*. It also removes a monomer from the monomer-set if it does not represent the most similar monomer for any block in *Blocks*(*Centromere, Monomers*).

Before launching the next iteration, MonomerGenerator recomputes the sequence of each monomer in the monomer-set by substituting it with the consensus of all blocks resolved by this monomer. In the resolved centromere, the *M*-consensus coincides with each monomer *M* in the generated monomer-set. Even though the final monomer-set is not guaranteed to be *Length-uniform*, it is not an issue for human centromeres, since most monomers in the human genome have a rather conserved length of ≅171 (Table 1). Supplementary Note ‘Pseudocode and complexity analysis for MonomerGenerator and HORDecomposer’ presents the pseudocode and complexity analysis for MonomerGenerator.

**Table 1.** Information about the MonomerGenerator results on cenX

Iter.	No. of resolved blocks	No. of unres. blocks	No. of non-mon. blocks	| largest comp |	radius	sep.	monomer-length
0	0	18 108	37	1507	8	32	171
1	1507	16 601	37	1506	8	47	171
2	3015	15 093	36	1505	6	35	171
3	4521	13 587	37	1501	9	26	171
4	6023	12 085	37	1499	7	44	171
5	7525	10 583	37	1500	7	30	171
6	9029	9079	37	1499	7	23	171
7	10 533	7575	37	1498	9	42	167
8	12 036	6072	37	1497	8	30	171
9	13 536	4572	37	1496	6	39	186
10	15 035	3073	37	1494	8	31	167
11	16 535	1573	37	1490	9	25	169
12	18 032	76	37	8	1	14	168
13	18 040	68	37	5	0	17	171
14	18 045	63	38	3	0	22	171
15	18 048	60	39	3	1	9	169
16	18 052	56	39	3	1	24	163
17	18 055	53	39	2	0	9	168
18	18 057	51	39	2	0	9	167
19	18 059	49	40	2	0	12	167
20	18 061	47	40	2	0	9	171
21	18 063	45	39	2	3	11	171
22	18 065	43	39	2	4	21	171
23	18 067	41	38	1	–	–	–

Each row corresponds to an iteration of the algorithm. At each iteration, the consensus of the blocks in the largest connected component in the block-graph is added to the monomer-set. At the 0th iteration, the monomer-set consists of the single *ConsensusMonomer*. The first three columns show the number of resolved, unresolved and non-monomeric blocks after running String Decomposer on the monomer-set at the corresponding iteration. In this Table, separation of a monomer generated at each iteration refers to the minimum distance to the previously generated monomers (rather than all generated monomers).

**Identification of hybrid monomers.** Given a string, we refer to a string formed by its first (last) *i* nucleotides as its *i-prefix* (*i*-suffix). We refer to a *hybrid monomer* formed by concatenating the *i*-prefix of a monomer *X* and the *j*-suffix of a monomer *Y* as the *X*(*i*)+*Y*(*j*), or simply *X *+* Y*, when omitting the indices *i* and *j* does not cause confusion. Hybrid monomers, albeit relatively infrequent, have been identified in several human centromeres (Dvorkina *et al.*, 2020). For each infrequent monomer *M*, MonomerGenerator identifies the most similar hybrid candidate generated by a pair of frequent monomers (*X*, *Y*) and reports *M* as a hybrid monomer if *div*(*M*, *X + Y)* does not exceed *MaxHybridDivergence* (default value 1%).

**Shifted monomer-set.** A unit of a tandem repeat is defined up to a *cyclic shift*. For example, AGGT, GGTA, GTAG and TAGG represent four cyclic shifts for a tandem repeat _**…**_AGGTAGGTAGGT_**…**_. However, in the case of a nested tandem repeat, the situation is more complex. For example, consider a nested tandem repeat _**…**_AGGTAACTTGGTAGGTAACTTGGT_**…**_, formed by three similar ‘monomers’ AGGT, AACT and TGGT (organized into a ‘HOR’ AGGTAACTTGGT). Shifting the starting positions of these monomers by two nucleotides results in a new monomer-set: GTAA, CTTG and GTAG. Note that the shifted monomers do not represent cyclic shifts but rather hybrids of the original monomers. Moreover, information about the original monomer-set is not sufficient for generating the shifted monomer-set in the case of a centromere with multiple HORs since information about the entire centromere is required to generate the shifted monomer-set.

Unfortunately, various studies of human centromeres used monomers with varying shifts (Bzikadze and Pevzner, 2020; Shepelev, 2015; Uralsky, 2019; Miga, 2020), making it difficult to compare the results and emphasizing the importance of selecting the standard representation of human monomers. To facilitate the comparison of the arbitrary monomer-sets (possibly with different shifts) MonomerGenerator has the MonomerGraph module that, given a monomer-set and a centromere, generates a shifted monomer-set.

**Monomer-graph.** Given a string *Centromere* and a monomer-set *Monomers*, StringDecomposer transforms it into a string *monoCentromere* over the alphabet of monomers and the ‘?’ symbols that represent non-monomeric blocks (Dvorkina *et al.*, 2020). A directed *monomer-graph* is constructed on a vertex-set of all monomers and the edge-set formed by all pairs of consecutive monomers in *monoCentromere.* The weight of an edge (*M, M′*) in the monomer-graph is defined as the number of times the monomer *M′* follows the monomer *M* in *monoCentromere.*

Given a monomer-graph constructed for monomer-set *Monomers*, MonomerGraph generates a new *i-shifted monomer-set Monomers*(*i*) by shifting the start of all monomers by *i* nucleotides. Each edge (*M, M′*) in the monomer-graph corresponds to a shifted monomer *M+M′* formed by concatenating the *i*-suffix of *M* with the *j*-prefix of *M′*, where *j* = |*M′*|-*i.* However, since different edges may result in identical (or similar) shifted monomers, we merge two shifted monomers into a single one if the divergence between them does not exceed *maxResolvedDivergence*/2 threshold. MonomerGraph also constructs the monomer-graph on shifted monomer by generating an edge between shifted monomers *M+M′* and *M′+M″* for each triple of consecutive monomers *M, M′*, *M″* in the monocentromere (the weight of this edge equals to the number of such triples).

**Identifying non-monomeric regions.** Most centromeres contain non-monomeric segments (e.g. Alu and LINE repeats) as well as highly diverged and truncated monomers that MonomerGenerator classifies as non-monomeric blocks. Supplementary Note ‘Identifying non-monomeric regions’ describes CentromereDecomposer—an extension of StringDecomposer that adds these non-monomeric regions as additional strings to the initial monomer-set, and generates a new string decomposition that takes into account these new non-monomeric strings. For example, CentromereDecomposer identified Alu repeats and partial monomers of length 113 in cen8 (Longsdon *et al.*, 2020; Supplementary Note ‘Non-monomeric regions in human centromeres’).

**HOR inference problem.** Despite four decades of HOR studies, we are not aware of a computational definition of a HOR that would allow one to rigorously derive all HORs in the human genome. Although Paar *et al.* (2005), Alkan *et al.* (2007) and Sevim *et al.* (2016) described various HOR inference heuristics (ColorHOR, HORdetect and Alpha-CENTAURI, respectively), these studies have not specified what is the exact objective function of these algorithms and have not formally defined the concept of a HOR. As a result, most attempts to derive HORs were based on manual effort rather than HOR inference algorithms, e.g. (McNulty and Sullivan, 2018) listed 36 human HORs, while Shepelev *et al.* (2015) listed 66 human HORs. Below we formulate the HOR Inference Problem, describe a simple greedy algorithm for its solution, and infer ∼100 frequent as well as ∼500 infrequent human HORs. We further introduce a concept of a superHOR and describe the decomposition of centromeres in superHORs.

Even though previous studies defined a HOR as a nucleotide sequence (such as DXZ1 HOR for cenX), we define a HOR as an arbitrary string in the monomer alphabet, moreover, a monomer may be repeated multiple times within a HOR (for example, this happens for HORs in human centromeres 4, 18, 20, 21). We argue that defining a HOR as a string in a monomer alphabet is a computationally more elegant and scalable approach that enables intra- and inter-species HOR comparison.

We denote the length of a string *S* as |*S*|, the number of elements in a set *A* as |*A*| and the total length of strings in a string-set *Strings* as *length*(*Strings*). Given a string-set *Strings*, an arbitrary concatenate of strings from this set is called a *String*-word. For example, if *Strings* = {AB, CD, BD}, ABBDCDAB is a *Strings*-word. We refer to the total number of strings from *Strings* that form a *Strings-*word *w* as *orbit*(*w*). For a *Strings*-word *w* = ABBDCDAB, |*w*| = 8 and *orbit*(*w*) = 4.

A *Strings*-word *w* is called a *Strings*-decomposition of a string *S* if *w *=* S*. The score of this *Strings*-decomposition *score*(*Strings, w*) is defined as *orbit*(*w*)+*length*(*Strings*). Given a string *S*, a string-set *Strings* is called *S*-*minimal* if there exists a *Strings*-decomposition *w* of *S* that minimizes *score*(*Strings, w*) over all string-sets *Strings* and over all *Strings*-decompositions of *S*. The elements of the *S*-minimal string-set are called *HORs.* We formulate the following HOR inference problem and note that it may have multiple solutions.

**HOR** Inference **Problem.**

**Input.** A string *S*.

**Output.** An *S*-minimal string-set *Strings.*

**String substitutions.** A string *S* over an alphabet *A* defines an *A*-decomposition *w* of *S* with *score*(*A, w*) = |*S*|+|*A*|. Given a substring *h* of a string *S*, we define *count_S_*(*h*) as the number of non-overlapping occurrences of *h* in *S*. There may be multiple ways to select *count_S_*(*h*) non-overlapping occurrences of *h* in *S*, e.g. *count*_AABBCAAAD_(AA) = 2 and there are two ways to select two non-overlapping occurrences of AA in AABBCAAAD: AABBCAAAD and AABBCAAAD. HORDecomposer selects the set of the ‘leftmost’ occurrences, i.e. AABBCAAAD over AABBCAAAD.

A string is called a *monorun* if it is made of a single symbol. A substring of a string is called a *run* if it is a maximal monorun, i.e. is not a substring of another monorun. For example, ABBCAAAD has two runs of A. The *run-length encoding* of a string *S* (denoted as S*) is defined as the substitution of each run of a symbol X of length *n* by an expression X^*n*^ that we count as a single symbol. For example, the run-length encoding of *S* = ABBCAAAD is S* = AB^2^CA^3^D, |*S*| = 8 and |S*| = 5.

Given a substring *h* of a string *S*, we define its *h-substitution* as a string *S*(*h*) resulting from substituting each of *count_S_*(*h*) non-overlapping occurrences of *h* in *S* by a new symbol *h*. For example, if *h*=AB, *g*=EGF and *S* = CABDEGFABEGFBDEGFABDEGFDABABDD with |*S*|=30, *S*(*h*) = ChDEGFhEGFBDEGFhDEGFDhhDD *=* ChDEGFhEGFBDEGFhDEGFDh^2^D^2^ and *S*(*g*) = CABDgABgBDgABDgDABABDD. Note that |*S*(*h*)| = 25, and |*S*(*h*)*| = 23, while |*S*(*g*)| = 22 and |*S*(*g*)*| = 21.

Adding *h* to the alphabet *A* forms a string-set *Strings* with |*A*|+1 elements and *length*(*Strings*) = |*A*|+|*h*|. The *h*-substitution operation results in a *Strings*-decomposition *w* of *S*(*h*) *with orbit*(*w*) = |*S*|-*count_S_*(*h*)*(|*h*|-1), *score*(*Strings, w*) = *orbit*(*w*) + *length*(*Strings*) = *(*|*S*|—*count_S_*(*h*)*(|*h*|—1)) + (|*A*| + |*h*|) = *score*(*A, w*) - *count_S_*(*h*)*(|*h*|-1) + |h|.

Thus, a sensible greedy strategy for solving the HOR Inference Problem is to find a substring *h* of a string *S* maximizing *count_S_*(*h*)*(|*h*|-1), perform the *h*-substitution and iterate. Below we describe the HORDecomposer algorithm that uses a slightly modified greedy strategy to generate an approximate solution of the HOR decomposition problem.

**Heavy substrings.** A substring *h* of a string *S* is called *recurrent* if *count_S_*(*h*) exceeds a threshold *MinCount* (default value *MinCount* = 5). A string is called *short* if its length does not exceed a threshold *MaxLength* (the default value is 30). Below we limit attention to short recurrent strings *h* and consider their *h*-substitutions. We define the weight *weight*(*S, h*) of a substring *h* as |S*|-|*S*(*h*)*|. A string *h* is called *heavy* if its weight exceeds a threshold *MinWeight* (default value *MinWeight* = 5).

**HORDecomposer algorithm.** A string is called *non-trivial* if it consists of at least two different symbols. We define a HOR in a string *S* as its recurrent heavy non-trivial substring *h* that minimizes run-length encoding of h-substitution of *S* over all recurrent heavy non-trivial substrings with at least one symbol (monomer) from the initial string *S* (ties are broken arbitrarily). The restriction that a new HOR has to include at least one monomer implies that we do not consider HORs formed by the previously constructed HORs (such HORs will be classified as superHORs at the follow-up stage). The HORDecomposer algorithm iteratively selects a heavy HOR *h* at each step, performs the *h*-substitution, and stops when there are no heavy HORs left. The resulting string is called the *HOR decomposition* of the string *S* (Fig. 2). Supplementary Note ‘Pseudocode and complexity analysis for MonomerGenerator and HORDecomposer’ presents the pseudocode for HORDecomposer.

Below we illustrate how HORDecomposer with parameters *MaxLength* = 30*, minCount* = 1 and *minWeight* = 10 works on a string *S*=CABDEGFABEGFBDEGFABDEGFDABABDD. HORDecomposer first selects a HOR a = EGF and transforms *S* into *S′ = S*(*a*) = CABDaABaBDaABDaDABABDD. Afterward, it selects a HOR b = AB, resulting in a string *S″ = S′*(b) = CbDabaBDabDaDbbDD. Afterward, it selects a HOR c = Da, resulting in a string *S‴ = S″*(c) = CbcbaBcbcDbbDD = CbcbaBcbcDb^2^D^2^. Note that HORDecomposer does not select d = bc as a HOR at the follow-up step since it does not contain monomers from the initial string *S.* Instead, this string will be identified as a superHOR as described below.

**Frequency of HORs.** A HOR in *HORDecomposition* = *HORDecomposition*(*Monocentromere, HORs*) is classified as *frequent* if its count exceeds the threshold |*HORDecomposition*|/*HORFreqCeiling* (default value *HORFreqCeiling =* 40), and *infrequent*, otherwise. An infrequent HOR is classified as *rare* if its count does not exceed a threshold *rareHORCount* (default value *rareHORCount* = 10).

**superHORs.** Each element in the HOR decomposition has a form *H^n^*, where *H* is a HOR and *n* is its *degree*, i.e. the number of tandem repeats of this HOR starting at a given position in a monocentromere (like in the HOR decomposition CbcbaBcbcDb^2^D^2^). To derive a *superHORs decomposition*, we ignore all degrees in the HOR decomposition (e.g. a string CbcbaBcbcDb^2^D^2^ is transformed into CbcbaBcbcDbD) and apply the HORDecomposer algorithm to the resulting string (albeit with the changed default parameters *MinCount = 2*, and *MinWeight = 2*). The resulting HORs are classified as *superHORs* (e.g. a superHOR bc in CbcbaBcbcDbD; Fig. 2). An example of a string ga^3^ga^15^ga^6^ga^4^ga^5^ga^39^ (a substring of the HOR decomposition of cenX) explains why we ignore the degrees in the definition of a superHOR. Indeed, g^n^a^m^ (for all possible values of *n* and *m*) is a compact representation of all six strings ga^3^, ga^15^, ga^6^, ga^4^, ga^5^ and ga^39^.

**Modifying HORDecomposer to incorporate known HORs.** Previous studies manually inferred some human HORs that we refer to as *canonical HORs* (McNulty and Sullivan, 2018; Shepelev *et al.*, 2015). For example, the canonical HOR for cenX is represented by a 12-monomer HOR (shown in the first row in Table 2), while the canonical HOR for cen6 is represented by an 18-monomer HOR (we represent it as a HOR *b* formed by concatenating a 14-monomer HORs *a* and 4 monomers M, N, O, P; see Supplementary Note ‘HOR and superHOR decomposition of cen6 and cen8’). Since the HORDecomposer is ‘blind’ with respect to canonical HORs, it may not include them in the list of inferred HORs (like in the case of cen6). However, to provide consistency with previous studies, it may be beneficial to force HORDecomposer to include canonical HORs. Supplementary Note ‘Modifying HORDecomposer to incorporate canonical HORs’ describes how HORDecomposer incorporates canonical HORs.

**Table 2.** Eight HORs in cenX (top), the HOR decomposition of cenX into these HORs and monomers (middle), and the superHOR decomposition of cenX into eight superHORs ada, BAf, af, ahKJ, ag, eah, eab and EDCBAf (bottom)

HOR name	HOR length	HOR	Count	Weight	Run-length
a	12	GFDECBALKJIH	1482	17 789	343
b	19	aGFDECBA	20	1611	219
c	16	KJIHa	18	1527	163
d	22	bLKJ	8	1482	136
e	3	NIH	8	1474	120
f	17	Lc	9	1469	109
g	17	Gc	8	1468	101
h	20	bL	7	1459	94
HOR decomposition		fa^114^da^180^GFECBAfa^33^f^3^a^2^fa^59^hKJBAfa^144^ga^3^ga^15^ga^6^ ga^4^ga^5^ga^39^bebea^12^hea^7^hea^18^hea^7^hea^17^hea^7^bea^239^bFED CBAfa^223^ga^3^ga^5^da^10^d^4^a^10^dada^127^b_LINE_ca^133^GEDCBAfa^21^hKJ			
superHOR decomposition		fadaGFECBAfafahKJBAfagabebeaheabFEDCBAf agadab_LINE_caGEDCBAfahKJ			

Powers in the HOR decomposition represent the length of a run, i.e. a^117^ stands for a_**…**_a repeating 117 times. Different colors represent different superHORs. The length of the run-length encoding of the HOR (superHOR) sequence for cenX is 94 (31).

**HOR-graph.** Let *Monomers, HORs* and *Monocentromeres* be the set of all monomers, *HORs* and monocentromeres in a genome, respectively. We construct an undirected *HOR-graph* with the vertex-set *HORs* and the edge-set formed by all pairs of HORs that share at least a single monomer (Fig. 2). We refer to a connected component of the HOR-graph as a *HOR-component*, to the most frequent HOR in each HOR-component as the *primary HOR* (ties are broken arbitrarily), and to all other HORs in a HOR-class as *secondary HORs* (also referred to as structural variants of HORs or *HOR StVs*, Miga *et al.*, 2020). Interestingly, some monomers in primary HOR for centromeres 4, 18, 20, 21 are repeated multiple times within the HORs. In most cases (15 out of 23), primary HORs in the HOR-graph of the human genome correspond to the canonical HORs inferred in previous studies (see Results, Table 3).

**Table 3.** Information about monomers and HORs inferred by CentromereArchitect on all human centromeres. Each row represents information about the alpha satellite array on a single chromosome. The second (third) column shows the number of frequent (hybrid) monomers generated by MonomerGenerator for each chromosome. The fourth column shows the total number of monomers generated by MonomerGenerator including frequent, hybrid, and infrequent monomers. The fifth (six) column shows the maximum radius (minimum separation ratio) for frequent monomers from the corresponding chromosome. Rows with separation ratios exceeding (not exceeding) 1 are highlighted in green(red). The seventh (eighth, ninth) column shows the total number of distinct HORs (frequent HORs, H-blocks) for each chromosome. The tenth column shows the most frequent HOR in each chromosome and their frequencies (chromosomes, where most frequent HOR is equal to canonical HOR, are shown in bold).

Chr	No. of freq mn-s	No. of hybr mn-s	Tot. mn-s	Max rad-s	Min. Sep-Ratio	No. of HORs	No. of freq. HORs	No. of *H*-bl-s	Most freq. HOR (#H-bl-s)
1	10	7	22	20	0.1	161	5	3874	12-mer(230)
2	4	0	9	13	0.615	37	7	2816	**4-mer(1348)**
3	17	0	23	9	1.5	13	5	532	**17-mer(312)**
4	17	3	22	13	0.833	44	9	1616	**19-mer(692)**
5	8	5	14	20	0.1	32	8	1704	4-mer(607)
6	18	1	19	10	1.286	7	2	953	**18-mer(643)**
7	6	1	14	17	0.765	12	2	3108	**6-mer(2852)**
8	11	1	12	10	1.857	6	3	1517	7-mer(646)
9	9	5	23	13	0.615	45	8	2264	11-mer(578)
10	8	6	36	12	1.11	39	6	1753	6-mer(585)
11	5	1	13	13	1	8	2	3898	**5-mer(3642)**
12	8	5	20	16	0.562	38	5	1956	**8-mer(1083)**
13	10	1	14	10	1.375	4	3	1323	7-mer(698)
14	8	1	12	18	1	12	1	1822	**8-mer(1701)**
15	12	0	16	10	1.125	11	6	486	15-mer(290)
16	10	0	16	20	0.1	15	4	1239	**10-mer(952)**
17	16	2	43	14	1.5	26	4	1567	**16-mer(985)**
18	11	2	20	12	0.89	42	4	2868	**12-mer(896)**
19	6	2	26	20	0.1	86	11	4421	4-mer(757)
20	15	0	17	13	0.615	13	4	836	**16-mer(628)**
21	10	1	14	10	1.375	5	5	185	**11-mer(151)**
22	8	1	13	18	1	14	2	2063	**8-mer(1819)**
X	12	2	14	9	1.625	8	1	1489	**12-mer(1444)**
**Tot.**	220	33	375	20	0.1	671	107	44 290	–

## 3 Results

### 3.1 Generating the monomer-set for cenX

Table 1 presents information about 23 monomers inferred by MonomerGenerator on cenX. Twelve (eleven) of these monomers are frequent (infrequent) and all infrequent monomers but two are rare.

**Fig. 3.**
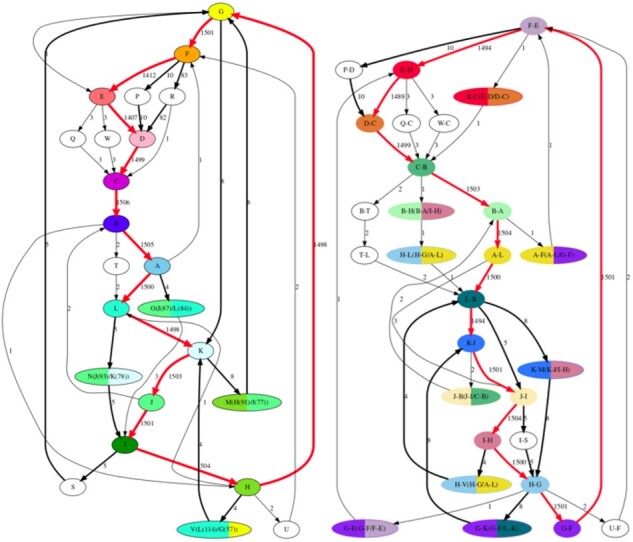
The monomer-graph for cenX for the initially generated monomers (left) and shifted monomers (right). The red cycle corresponds to the most frequent 12-monomer HOR in cenX (DXZ1). Edges with a weight exceeding 3 are shown in bold. (Left) The monomer-graph has 23 vertices and 41 edges. (Right) After shifting by 94 nucleotides, the monomer-graph has 28 vertices and 44 edges. Vertices corresponding to 12 frequent monomers are colored by 12 different colors. Hybrid monomers *X*/*Y* are represented by bicolored vertices, where two colors correspond to frequent monomers *X* and *Y*. A vertex labeled as X–Y corresponded to a shift of the initially generated monomers X and Y

We follow Shepelev *et al.* (2015) in the selection of the cyclic shift for the initial alpha-satellite consensus that defines the *reference monomers* inferred in previous studies (Supplementary Note ‘Consensus monomer and reference monomers’). Monomer alignments revealed that the reference monomers are shifted by 94 nucleotides as compared to the monomers generated by the MonomerGenerator. After shifting by 94 nucleotides and merging similar monomers, we generated 12 frequent monomers and sixteen infrequent monomers (Fig. 3). The frequent monomers correspond to reference monomers that form the abundant DXZ1 HOR in cenX (Waye and Willard, 1985). The infrequent monomers include 9 hybrid monomers and 7 variants of frequent monomers with large indels. 11 out of 16 infrequent monomers are rare.

Supplementary Note ‘Comparing monomers generated by MonomerGenerator with the reference monomers’ compares the reference monomers with the monomers generated by MonomerGenerator. The frequent monomers are the first to be generated by MonomerGenerator (Table 1) and are very similar to the corresponding reference monomers. Three frequent monomers coincide with the reference monomers, two monomers have an insertion of a single nucleotide, one monomer has a single mismatch, and six monomers have a few small gaps either at the start or at the end. A few mismatches can be explained by inaccuracies in the previously derived reference monomers and/or centromere polymorphism across the population. Indels at the start and end of monomers are due to minor inconsistency of the shift selection between some reference monomers and frequent monomers.

Below we discuss MonomerGenerator results on cenX (Supplementary Note ‘Monomer inference for cen6 and cen8’ benchmarks MonomerGenerator on cen6 and cen8). One of the cenX monomers inferred by MonomerGenerator [*G-K(G-F/L-K)* in Supplementary Note ‘Comparing monomers generated by MonomerGenerator with the reference monomers’] represents a monomer *M* that corresponds to a K(68)+F(103) hybrid of the frequent monomers K and F identified in Bzikadze and Pevzner (2020) and Dvorkina *et al.* (2020**)**. The locations of the *M*-blocks are flanked by J-blocks on the left and G-blocks on the right. Since the canonical 12-monomer HOR in cenX is ABCDE**FG**HI**JK**L, the K + F hybrid has likely arisen from a deletion in ABCDE**FG**HI**JK**LABCDE**FG**HI**JK**L that removed a suffix of an F-block and a prefix of a K-block. Similarly, the *K-M(K-J/I-H)* **(***H-V(H-G/A-L*) monomer is a G(129) + I(42) *(*J(112) + E(55)) hybrid that has likely arisen from a deletion in ABCDEF**G**H**I**JKL (ABCDEFGHI**J**KLABCD**E**FGHIJKL). Also, MonomerGenerator inferred 5 rare hybrids in cenX.

### 3.2 Inferring HORs and superHORs for cenX

The *monolength* of the centromere is defined as the total number of (monomer) blocks in its monocentromere. For example, if one ignores infrequent monomers, cenX, cen8 and cen6 are written in the alphabets of 14 (12 + 2 hybrid), 12 (11 + 1 hybrid) and 16 (15 + 1 hybrid) frequent monomers, respectively, and have monolengths 18 145, 12 251 and 16 315, respectively. For monocenX, HORDecomposer infers 8 HORs (single frequent and 7 rare HORs) and generates a HOR decomposition of cenX into a string with the length of its run-length encoding equal to 94 (Table 2). It further infers seven superHORs and generates a superHOR decomposition of cenX of length 31. Interestingly, many superHORs occupy long contiguous segments of the centromere, providing insights into centromere evolution. We refer to each symbol *H* in the HOR decomposition of a centromere as an *H-block*. Table 3 summarizes the number of different HORs and *H*-blocks for each centromere. Supplementary Note ‘HOR and superHOR decomposition of cen6 and cen8’ presents HOR decompositions of cen6 (7 HORs and 11 superHORs) and cen8 (6 HORs and 9 superHORs).

### 3.3 Generating monomers and HORs for the entire set of live human centromeres

Previous studies of human alpha satellite HORs were based on the centromeric *Reference Models (RMs)* incorporated in the hg38 assembly of the human genome. These models are collections of all Sanger reads that match a certain HOR, (combined into a single sequence by the stochastic Markov process) that do not represent the correct sequences of centromeres (Miga *et al.*, 2014; Rosenbloom *et al.*, 2014). Thus, it is not surprising that our study revealed a much larger set of human HORs.

Generation of RMs includes two steps: (i) inferring HOR consensus sequences from a set of Sanger reads and (ii) generating the stochastically simulated alpha satellite arrays from the read-set for each HOR using the reconstructed consensus HOR as a seed. The algorithm for HOR reconstruction and the method of anchoring them in the simulated assembly remain unpublished, but the protocol for generating an RM using a seed sequence was published in Miga *et al.* (2014). Based on RMs, Shepelev *et al.* (2015) reconstructed 66 human HORs, the largest human HOR-set reconstructed so far. Of these, 18 unique models represent 22 live centromeres of autosomes, as chromosomes 13/21, 14/22 and 1/5/19 share the same live reference models. Two additional models represent live centromeres of sex chromosomes. Sevim *et al.* (2016) have used this set of HORs to annotate human PacBio reads and Uralsky *et al.* (2019) have used it to extend the HOR classification in a single alpha satellite suprachromosomal family (see Supplementary Note: ‘HOR hierarchy’) by manually curating it and adding a new class of low-copy divergent HORs. CentromereArchitect inferred 107 frequent, 566 infrequent and 327 rare HORs.

Table 3 presents results of MonomerGenerator on all human centromeres (see Supplementary Note ‘Information about human centromeres’). In total, MonomerGenerator inferred 220 frequent, 33 hybrid, 155 infrequent and no rare monomers in human centromeres.

**Fig. 4.**
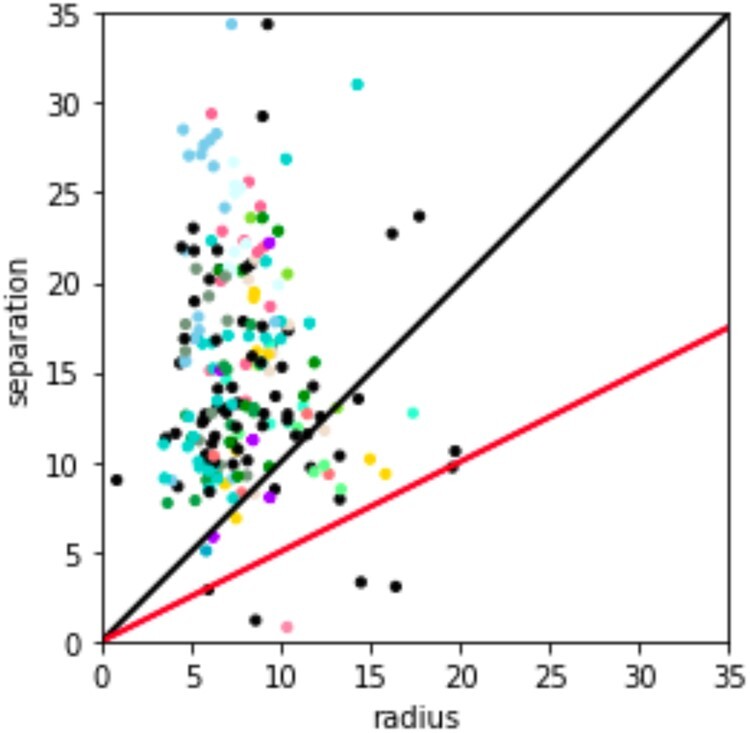
Information about the radius (*x*-axis) and separation (*y*-axis) of all 220 frequent human monomers. Only for 21 out of 220 frequent human monomers, located below the black line *separation*=*radius*, the separation ratio is below 1 (only 4 of them, located below the red line *separation *=* *0.5*radius, have separation ratio below 0.5). Each circle corresponds to a single frequent monomer. For a clearer picture, the circles have a slight random offset. The colors of the points correspond to specific chromosomes except for black circles that represent monomers shared between several chromosomes. The light pink circle with radius 10 and separation 1 corresponds to centromere 1

Figure 4 presents the distribution of radius and separation of all frequent human monomers. We used the separation ratio to assess the quality of the generated monomers (monomers with high separation ratio rarely result in ambiguous assignments of their *M*-blocks) and analyzed *separationRatio*(*Centromere*) defined as the minimum separation ratio of all monomers from this centromere. For example, cen8 has the highest *separationRatio*(cenX)=1.9, while cen1 has the smallest *separationRatio*(cen1)=0.1.

Twelve human centromeres have separation ratios exceeding 1. Most centromeres with a separation ratio below 1 contain monomers with an unusually high radius that may reflect ‘old’ monomers that significantly diverged from their consensus. Also, nearly all centromeres with a separation ratio below 1 (except for cen7 and cen13) contain monomers shared with other centromeres. The radius of such shared monomers may be larger than the radius of other monomers because they are formed by ‘submonomers’ from various centromeres (with slightly different consensuses) that were clustered together by MonomerGenerator. Further sub-clustering of monomers into monomer subfamilies may be a sensible approach to address such over-clustering (see Supplementary Note ‘Generating submonomers for cenX’).

### 3.4 Cross-chromosome HOR and monomer comparison

Alpha satellite HORs present a complex hierarchy of sequences with different levels of divergence between different HORs and between copies of the same HOR within a centromere (Alexandrov *et al.*, 2001; Bzikadze and Pevzner 2020; McNulty and Sullivan 2018; Miga, 2020; Shepelev *et al.*, 2015; Uralsky *et al.*, 2019). Supplementary Note ‘HOR hierarchy’ describes different levels of this hierarchy.

Out of 671 total HORs in the live centromere arrays of the human genome, only six are shared between several chromosomes. These *shared HORs* consist of monomers that are shared between chromosomes 1, 5, 16 and 19. For simplicity, we refer to the monomer D1/5/16/19 as D, and monomer E1/5/16/19 as E, and monomer F1/5/16/19 as F. Six shared HORs include: FDF in chromosomes 1, 5 and 19; DEDE, DFDE, FDE, FDFD, FDFDF in chromosomes 1 and 19.

The HOR-graph generated for the set of all 671 human HORs consists of 15 HOR-components with sizes ranging from 6 to 287. All but 5 HOR-components represent HORs from a single chromosome. HOR-components of size 287 (92, 89, 26 and 9) combine HORs that originated from chromosomes 1,5,16,19 (2,18,20; 4,9; 14,22; and 13,21).

Each row represents information about the alpha satellite array on a single chromosome. The second (third) column shows the number of frequent (hybrid) monomers generated by MonomerGenerator for each chromosome. The fourth column shows the total number of monomers generated by MonomerGenerator including frequent, hybrid and infrequent monomers. The fifth (six) column shows the maximum radius (minimum separation ratio) for frequent monomers from the corresponding chromosome. Rows with separation ratios exceeding (not exceeding) 1 are highlighted in green(red). The seventh (eighth, ninth) column shows the total number of distinct HORs (frequent HORs, *H*-blocks) for each chromosome. The tenth column shows the most frequent HOR in each chromosome and their frequencies (chromosomes, where most frequent HOR is equal to canonical HOR, are shown in bold).

## 4 Discussion

Recent advances in long-read sequencing technologies and genome assembly algorithms opened new horizons for human centromere genomics. For the first time, structural and evolutionary studies of human alpha satellite arrays can be based on complete centromere assembly rather than individual reads or *satellite reference model*s (Miga *et al.*, 2014). We introduced the computationally rigorous definitions of monomers and HORs and developed CentromereArchitect, the first centromere annotation tool that contains MonomerGenerator for inferring monomers and HORDecomposer for inferring HORs. Applying CentromereArchitect to the nearly complete human genome assembly by the T2T consortium resulted in the first comprehensive database of human monomers and HORs in live centromeres. The development of CentromereArchitect is an important prerequisite for future centromere research, including population-wide analysis of human monomers and HORs, evolutionary studies of centromeres across primates and other species, biomedical studies of diversity of human centromere sequences and their associations with genetic diseases, and other important applications.

Since both MonomerGenerator and HORDecomposer are heuristic algorithms, we benchmarked their running time performance on real data. Running MonomerGenerator on cenX takes less than an hour of clock time when executed in 30 threads. The most computationally intensive stage is running StringDecomposer to generate the decomposition of centromeres into blocks. Running MonomerGenerator on all live human centromeres takes approximately a week of clock time. Such seemingly extensive runtime is acceptable because MonomerGenerator needs to be done once for a genome. However, the computational challenge of optimizing MonomerGenerator will become prominent as complete assemblies of multiple human genomes emerge. HORDecomposer does not present a computational bottleneck as it takes minutes to run on all live centromeres.

CentromereArchitect assumes that the quality of the centromere assemblies is exceptionally high. Since live centromeres are extra-long tandem repeats, generating accurate centromere assemblies is a difficult computational challenge that was unresolved for almost two decades since the completion of the Human Genome Project (Bzikadze and Pevzner, 2020; Miga *et al.*, 2020; Nurk *et al.*, 2020, S.Nurk *et al.*, submitted for publication). However, the public release of the Telomere-to-Telomere assembly v1.0 (that is used in this paper) has been evaluated by the TandemQUAST tool (Mikheenko *et al.*, 2020). This evaluation showed no structural errors and no regions with deteriorated accuracy of base-calling. Ultimately, we will update the set of monomers and HOR decomposition as improved versions of the assembly become available.

Since there is only a single complete human genome assembly available to date, the selection of defaults for CentromereArchitect parameters is particularly challenging. Supplementary Note: ‘Parameters of CentromereArchitect’ describes our rationale for tuning these parameters.

Even though CentromereArchitect successfully extracted human monomers and HORs, it has certain limitations that we plan to address in a follow-up study and that are outlined below.

**Divergent monomeric and HOR layers.** Biologists distinguish between *homogeneous* HOR domains that the kinetochore binds to and that feature small divergence that does not exceed 10% (Uralsky *et al.*, 2019) and *divergent* HOR domains that are covered by very diverged HOR-blocks (more than 10% divergence) or formed by monomers that do not form well-defined HORs. Although CentromereArchitect successfully extracts monomers and HORs for homogeneous HOR domains, further algorithmic developments are needed to extend Centromere Architect to *divergent* HOR domains. The layers with divergent HOR domains are the oldest among all alpha satellite domains in the human genome and their annotation may help to provide insights into the development of centromeres in primates and understanding of the Centromere Paradox.

**Genome-wide submonomer detection.** Since some centromeres share very similar monomers (Uralsky *et al.*, 2019), MonomerGenerator typically over-cluster such shared monomers into a single cluster. Even though CentromereArchitect provides initial insights into submonomer detection (see Supplementary Note ‘Generating submonomers for cenX’) further developments are needed to optimize submonomer identification with the goal to subpartition all monomers with high separation ratios into submonomers.

**Diploid centromeres.** Although the T2T consortium generated the first nearly complete assembly of the effectively haploid CHM13 cell line, centromere assembly in diploid genomes remains an open problem (Cheng *et al.*, 2021). CentromereArchitect will face additional algorithmic challenges when applied to diploid human genome assemblies.

The HOR Decomposition Problem is closely related to the classical Data Compression Problem (Storer, 1987). Since centromeres are extra-long tandem repeats (with small variations between the repeat copies), the existing data compression algorithms can be applied to centromeres. Since MonomerGenerator clusters similar blocks into monomers, the monomer decomposition is lossy and irreversible. On the other hand, the HOR decomposition of a monocentromere is lossless and reversible. The HOR decomposition of the cenX monocentromere (18 145 blocks) results in a run-length encoding with only 147 characters (two orders of magnitude compression). Even though this encoding is rather short, more efficient encodings might exist.

We introduced computational definitions of a monomer (*M*-block), HOR (*H*-block), HOR-graph (HOR-component) and primary (secondary) HORs. Since some of these definitions differ from the previously introduced (and often only informally defined) concepts, Supplementary Note ‘Summary of centromeric building blocks’ provides intuition behind each of these concepts in the hope to establish a bridge with previous studies of centromeres.

## Supplementary Material

btab265_Supplementary_Data
