# Supplementary material for: CentromereArchitect: inference and analysis of the architecture of centromeres
Source: Bioinformatics. 2021 Jul 12;37(Suppl 1):i196–204. doi: 10.1093/bioinformatics/btab265 (PMC8336445; doi:10.1093/bioinformatics/btab265)
Supplement: btab265_Supplementary_Data [file btab265_supplementary_data.zip › btab265-suppl_data/Bzikadze.217.sup.marked.pdf]

Supplementary Notes for "CentromereArchitect: inference and analysis of the architecture of centromeres"

- Information about human centromeres
- Consensus monomer and reference monomers
- Constructing connected components of the block-graph
- Pseudocode and complexity analysis of MonomerGenerator and HORDecomposer
- Identifying non-monomeric regions
- Non-monomeric regions in human centromeres
- HOR and superHOR decomposition of cen6 and cen8
- Modifying HORDecomposer to incorporate canonical HORs
- Comparing monomers generated by MonomerGenerator with the reference monomers
- Monomer inference for cen6 and cen8
- Generating submonomers for cenX
- HOR hierarchy
- Parameters of CentromereArchitect
- Summary of centromeric building blocks

Supplementary Note: Information about human centromeres

Table SN:HC-1 contains coordinates of the (live) alpha satellite arrays in all human chromosomes in the public release v1.0 by the Telomere-to-Telomere consortium of the nearly complete assembly for the CHM13 cell line (https://github.com/nanopore-wgs-consortium/chm13#v10). These coordinates were manually extracted.

Chromosome	start	end
1	121 796 218	126 300 656
2	92 333 539	94 673 018
3	91 735 618	92 596 313
3	92 869 954	92 903 597
3	95 863 962	96 415 434
4	49 705 249	50 433 651
4	52 115 581	54 870 604
4	54 980 385	55 199 889
5	47 039 130	47 049 658
5	47 077 199	49 596 619
6	58 286 939	61 058 622
7	60 414 370	63 714 496
8	44 243 543	46 325 076
9	44 952 791	47 582 587

10	39 633 786	41 881 061
11	51 035 791	54 413 485
12	34 620 831	37 202 143
13	16 220 361	18 181 363
14	10 149 798	12 766 096
15	17 263 917	18 279 251
16	35 848 293	37 829 526
17	23 433 664	27 571 610
18	15 965 700	20 933 550
19	25 821 756	29 768 168
20	26 925 847	29 099 648
21	11 699 868	12 043 391
22	12 816 950	15 739 833
X	57 817 899	60 927 196

Table SN:HC-1. Coordinates of the alpha satellite arrays in all human chromosomes. Chromosomes 3 (4; 5) contains three (three; two) alpha satellite arrays that are separated by non-monomeric regions of lengths 274 kb and 2960 kb (1682 kb and 110 kb; 27 kb).

Supplementary Note: Consensus monomer and reference monomers

Consensus monomer. We use the *type A* monomer (cons_A_type_AJ131207|modified|12-àG|44 deleted) as a consensus alpha-satellite monomer in the human genome (Shepelev et al., 2015). The unspecified "N" nucleotides in this sequence were filled according to the majority votes in the nucleotide profile matrix (Shepelev et al., 2015), resulting in the following *ConsensusMonomer* sequence:

```
AATCTGCAAGTGGACATTTGGAGCGCTTTGAGGCCTATGGTGGAAAAGGAAATATCTTCACATAAA
AACTAGACAGAAGCATTCTCAGAAACTTCTTTGTGATGTGTGCATTCAACTCACAGAGTTGAACCT
TTCTTTTGATAGAGCAGTTTTGAAACACTCTTTTTGTAG
```

Reference monomers. We refer to monomers that are forming the alpha satellite arrays in centromeres 6, 8, and X as the *reference monomers* — 18 (15, 12) monomers for centromeres 6, (8, X). These monomers were extracted in a manual fashion (Dvorkina et al., 2020, also see https://github.com/ablab/stringdecomposer). We refer to the canonical 12-monomer cenX HOR as ABCDEFGHIJKL.

Supplementary Note: Constructing connected components of the block-graph

The block-set *Blocks*(*Centromere,Monomers*) for the entire human genome contains nearly 300,000 blocks. Since the brute-force construction of the block-graph (that computes the edit distance between all pairs of blocks) faces the running time and memory bottlenecks, we use

the following approach to speed-up its construction and substantially reduce its running time and memory footprint.

Given an arbitrary vertex *v* in the block-graph, we compute its distance to all other vertices and use this information to speed-up the construction of the connected component containing this vertex (referred to as *component*(*v*)). Given the ranked list of all vertices in the increasing order of distances from *v,* we quickly generate *component*(*v*) by starting with a single vertex *v* and gradually adding more vertices by scanning this list. Note that $d(w,u) < maxResolvedDivergence / 2$ for any two adjacent vertices *w* and *u* in the block-graph. Therefore, if a vertex *w* has already been added to *component*(*v*), each its neighbor *u* in the block-graph satisfies the triangle inequality $d(v,w)+maxResolvedDivergence / 2 > d(v,u)$. Therefore, we only need to analyze vertices with the distance below $d(v,w)+maxResolvedDivergence / 2$ while extending *component*(*v*) by neighbors of *w*. This observation greatly speeds-up the construction of the block-graph.

At each iteration of the algorithm for constructing the connected components of the block-graph, we randomly select a vertex *v* that does not belong to the already constructed components, construct *component*(*v*), remove all vertices of *component*(*v*) from further consideration, and iterate.

Supplementary Note: Comparing monomers generated by MonomerGenerator with the reference monomers

Table MonomersComparison compares monomers generated by MonomerGenerator with the reference monomers and annotates the hybrid monomers for cenX. Infrequent monomers *W-C*, *Q-C* likely represent diverged variants of the reference monomer *B* as they differ from *B* by large deletions of 19 and 33 nucleotides, respectively.

<table>
<tr><th>generated monomer</th><th>reference monomer</th><th>divergence with the reference monomer</th><th>number of occurrences in cenX</th></tr>
<tr><td colspan="4">frequent monomers</td></tr>
<tr><td>F-E</td><td>L</td><td>1.16</td><td>1412</td></tr>
<tr><td colspan="4">TCTATGTCGTTTTAGGAGAAGATATTTCCTTTTCCAACACAGTCCTCCAAGCCCGCTAAATAGCCACTTGCACATTGTAGAAAAAGTGTGTCAAAGCTGCGCTATCAAAGGGAAAGTTCAACTCTGTGAGGTGAATGCAAACATCCCAAAGAAGTTTCTGAGAATGCT--
|||||||||||||||||||||||||||||||||||||||||||||||||||||||||||||||||||||||||||||||||||||||||||||||||||||||||||||||||||||||||||||||||||||||||||||||||||||||||||||||||||||||||||--
TCTATGTCGTTTTAGGAGAAGATATTTCCTTTTCCAACACAGTCCTCCAAGCCCGCTAAATAGCCACTTGCACATTGTAGAAAAAGTGTGTCAAAGCTGCGCTATCAAAGGGAAAGTTCAACTCTGTGAGGTGAATGCAAACATCCCAAAGAAGTTTCTGAGAATGCTTC</td></tr>
<tr><td>E-D</td><td>A</td><td>1.76</td><td>1407</td></tr>
<tr><td colspan="4">TCCGTTTAGCTTTTAGGTGAAGATTATCCCGTTTCCAACGAAACCTTCAAAGAGGTCCAAATATCCCCTTGCGGATCCCACAGAAAGAGTGTTTCGAAACTGCTGTTTCAAA-GGAATCTTCAACTCTGTGAGTTGAATGCAATCATCACAAAGAAGTTTCTGACAATGCT
--||||||||||||||||||||||||||||||||||||||||||||||||||||||||||||||||||||||||||||||||||||||||||||||||||||||||||||||-||||||||||||||||||||||||||||||||||||||||||||||||||||||||||
--CGTTTAGCTTTTAGGTGAAGATTATCCCGTTTCCAACGAAACCTTCAAAGAGGTCCAAATATCCCCTTGCGGATCCCACAGAAAGAGTGTTTCGAAACTGCTGTTTCAAAAGGAATCTTCAACTCTGTGAGTTGAATGCAATCATCACAAAGAAGTTTCTGACAATGCT</td></tr>
<tr><td>D-C</td><td>B</td><td>0</td><td>1499</td></tr>
<tr><td colspan="4">TCTCTCTCGTCTTTCTGTGAAGATAAAGGAAAAGGCTTTCAGGCCTTTTCCACCACAGGCCTGAAAGCGCTCCAAATGTCCACTTGCAGATTCTGCCAAAAGAATATTTCAAAACTGCTCTATGAAAAGCAATGTTAAACTCTGTGGCTCGAACACAAACATCACAAAGCAGTTTCTGAGAAT
GCT
|||||||||||||||||||||||||||||||||||||||||||||||||||||||||||||||||||||||||||||||||||||||||||||||||||||||||||||||||||||||||||||||||||||||||||||||||||||||||||||||||||||||||||||||||||
|||
TCTCTCTCGTCTTTCTGTGAAGATAAAGGAAAAGGCTTTCAGGCCTTTTCCACCACAGGCCTGAAAGCGCTCCAAATGTCCACTTGCAGATTCTGCCAAAAGAATATTTCAAAACTGCTCTATGAAAAGCAATGTTAAACTCTGTGGCTCGAACACAAACATCACAAAGCAGTTTCTGAGAAT
GCT</td></tr>
<tr><td>C-B</td><td>C</td><td>0</td><td>1506</td></tr>
<tr><td colspan="4">TCAGTTTAGTTTTTCTGTGGAAATATTCCCGTTTCCAAAGAAATCTTCAAAGAGGTCCACGTATCCACTTACAGATTCTACAAAAAGACAGTTTCAAAACTGCTCCATCAAAAGGAGGGTTCAACTGTGTGACTTGAATGCAATCATCACTCAGAAGTTTCTGAGAATGCT
|||||||||||||||||||||||||||||||||||||||||||||||||||||||||||||||||||||||||||||||||||||||||||||||||||||||||||||||||||||||||||||||||||||||||||||||||||||||||||||||||||||||||||||
TCAGTTTAGTTTTTCTGTGGAAATATTCCCGTTTCCAAAGAAATCTTCAAAGAGGTCCACGTATCCACTTACAGATTCTACAAAAAGACAGTTTCAAAACTGCTCCATCAAAAGGAGGGTTCAACTGTGTGACTTGAATGCAATCATCACTCAGAAGTTTCTGAGAATGCT</td></tr>
</table>

B-A	D	0.58	1505
TCTCTTTAGTTTTTACGTGAACATATACCCGTTTCGAACGAAGGCCACCCAGTGGTCCAAATATCCACTTGCAGATTCTACAGAAAGAGTGTTTCGAACCTGAACTCTCAAAGGCAGGTTCATCTCTGCGAGTTAAATGCATTCATCATGAAGAACTTTCTCAGAGTGTT-<br>||||||||||||||||||||||||||||||||||||||||||||||||||||||||||||||||||||||||||||||||||||||||||||||||||||||||||||||||||||||||||||||||||||||||||||||||||||||||||||||||||||||||||-<br>TCTCTTTAGTTTTTACGTGAACATATACCCGTTTCGAACGAAGGCCACCCAGTGGTCCAAATATCCACTTGCAGATTCTACAGAAAGAGTGTTTCGAACCTGAACTCTCAAAGGCAGGTTCATCTCTGCGAGTTAAATGCATTCATCATGAAGAACTTTCTCAGAGTGTTT			
A-L	E	0.59	1500
TGTGTTTAGTTATGGGAAATTATTCCCGTTTCCAACGAAATCCTCAGAGAGCTCCAAATATCCACCTGCAGATTCTACCAAAGTGTATTTGGAAACTGCTCCATCAAAAGGCATGTTCAGCTCTGTGAGTGAAACTCCATCATCACAAAGAATATTCTGAGAATGCT<br>-|||||||||||||||||||||||||||||||||||||||||||||||||||||||||||||||||||||||||||||||||||||||||||||||||||||||||||||||||||||||||||||||||||||||||||||||||||||||||||||||||| -GTGTTTAGTTATGGGAAATTATTCCCGTTTCCAACGAAATCCTCAGAGAGCTCCAAATATCCACCTGCAGATTCTACCAAAGTGTATTTGGAAACTGCTCCATCAAAAGGCATGTTCAGCTCTGTGAGTGAAACTCCATCATCACAAAGAATATTCTGAGAATGCT			
L-K	F	0.58	1498
TCCGTTTGCCTTTTATATGAAGTTCCTTCCTGTACTACCGTAGGCCTCAAAGCAGTCCAAATCTCCATTTGCAGATTCTACAAAAAGAGTGATTCCAATCTGCTCTATCAATAGGATTGTTCAACTCCATGAGTTGAATGCCATCCTCACAAAGTCGTTTCTGAGAATGCT<br>|||||||||||||||||||||||||||||||.||||||||||||||||||||||||||||||||||||||||||||||||||||||||||||||||||||||||||||||||||||||||||||||||||||||||||||||||||| TCCGTTTGCCTTTTATATGAAGTTCCTTCCTATACTACCGTAGGCCTCAAAGCAGTCCAAATCTCCATTTGCAGATTCTACAAAAAGAGTGATTCCAATCTGCTCTATCAATAGGATTGTTCAACTCCATGAGTTGAATGCCATCCTCACAAAGTCGTTTCTGAGAATGCT			
K-J	G	0.58	1503
TCTATCTAGTTTTTATGTGAAGATATTTCCTTTTCCACCACAGGCCTCAAAGCCCTCCAAACGTCCACTTGCAGATTCTCGAAAAAGAGTGTTTCATAGCTGCTCTTTCAAA-GGAAAGTTCAACTCTGGGAGTTGAATACAAACATCACAAAGTAGTTTCCGAGAATGCT<br>||||||||||||||||||||||||||||||||||||||||||||||||||||||||||||||||||||||||||||||||||||||||||||||||||||||||||||||||-|||||||||||||||||||||||||||||||||||||||||||||||||||||||||| TCTATCTAGTTTTTATGTGAAGATATTTCCTTTTCCACCACAGGCCTCAAAGCCCTCCAAACGTCCACTTGCAGATTCTCGAAAAAGAGTGTTTCATAGCTGCTCTTTCAAAAGGAAAGTTCAACTCTGGGAGTTGAATACAAACATCACAAAGTAGTTTCCGAGAATGCT			
J-I	H	0	1501
TCTGTTTAGTTTTTATGTGAAGATGATCCCGTTTCCAGTGAAATCTTCAAAGAGGTCCACATATCCCCTTGCAGATTCCAAAGAAAGAGGGTTTCAAAACTGCTCCATCAGAAGGATTGTTCAACTCTGTGAGTTGAATGCAGTCATCGCAGAAAACTTTCTGAGAATGCT<br>||||||||||||||||||||||||||||||||||||||||||||||||||||||||||||||||||||||||||||||||||||||||||||||||||||||||||||||||||||||||||||||||||||||||||||||||||||||||||||||||||||||| TCTGTTTAGTTTTTATGTGAAGATGATCCCGTTTCCAGTGAAATCTTCAAAGAGGTCCACATATCCCCTTGCAGATTCCAAAGAAAGAGGGTTTCAAAACTGCTCCATCAGAAGGATTGTTCAACTCTGTGAGTTGAATGCAGTCATCGCAGAAAACTTTCTGAGAATGCT			
I-H	I	0.58	1504
TCTGTCTAGGTTTGATGTGAAGATATAGACGTTTCAAACGAAGGCTACAAAGTGGTCAAAATATACACTTGCAGATTCTACTACAAGGGTGTTGCAAACCTGAACTATCAAAGGAAGGTTCAACTCTGTGAGTTGAATACAAACATCACAAAGAATGTTCTGAGTTTGCT-<br>||||||||||||||||||||||||||||||||||||||||||||||||||||||||||||||||||||||||||||||||||||||||||||||||||||||||||||||||||||||||||||||||||||||||||||||||||||||||||||||||||||||||||-<br>TCTGTCTAGGTTTGATGTGAAGATATAGACGTTTCAAACGAAGGCTACAAAGTGGTCAAAATATACACTTGCAGATTCTACTACAAGGGTGTTGCAAACCTGAACTATCAAAGGAAGGTTCAACTCTGTGAGTTGAATACAAACATCACAAAGAATGTTCTGAGTTTGCTT			
H-G	J	0.6	1498
TCCGTTCAGTTATGGGAAGTTGATCCCGTTTCCAACGAAATCCTCAGAGAGGTCCAAATATCCCCTTGCAGATTCTACAAAACGTGTGTTTGGAAACTGCTCCATCATAACGAATGTTCAGCTCCCTGAGTTAAACTCCATCGTCACAAAGAATTTTCTGAGAGTGCT<br>-||||||||||||||||||||||||||||||||||||||||||||||||||||||||||||||||||||||||||||||||||||||||||||||||||||||||||||||||||||||||||||||||||||||||||||||||||||||||||||||||||||| -CCGTTCAGTTATGGGAAGTTGATCCCGTTTCCAACGAAATCCTCAGAGAGGTCCAAATATCCCCTTGCAGATTCTACAAAACGTGTGTTTGGAAACTGCTCCATCATAACGAATGTTCAGCTCCCTGAGTTAAACTCCATCGTCACAAAGAATTTTCTGAGAGTGCT			
G-F	K	0.58	1503
ACCGTCTGGTTTTTATATGAAGTTCTTTCCTTCACTACCACAGGCCTCAAAGCGGTCCAAATCTCCACTTGCAGATTCTACAAAAAGAGTGTTTGCAAACTGCTCTATCAAA-GGAATGTTCAACTCTGGGAGTTGAATGCAATCATCACAGAGCAGTTTCTGAGAATGCT<br>||||||||||||||||||||||||||||||||||||||||||||||||||||||||||||||||||||||||||||||||||||||||||||||||||||||||||||||||-|||||||||||||||||||||||||||||||||||||||||||||||||||||||||| ACCGTCTGGTTTTTATATGAAGTTCTTTCCTTCACTACCACAGGCCTCAAAGCGGTCCAAATCTCCACTTGCAGATTCTACAAAAAGAGTGTTTGCAAACTGCTCTATCAAAAGGAATGTTCAACTCTGGGAGTTGAATGCAATCATCACAGAGCAGTTTCTGAGAATGCT			
**infrequent monomers**			
G-K(G-F/L-K)	K+F	7.6+6.43	8
ACCGTCTGGTTTTTATATGAAGTTCTTTCCTTCACTACCACAGGCCTCAAAGCGGTCCAAATCTCCACTTGCAGATTCTACAAAAAGAGTGTTTGCAAACTGCTCTATCAA-AGGAATGTTCAACTCTGGGAGTTGAATGCAATCATCACAGAG-CAGTTTCTGAGAATGCT<br>|||||||||||||||||||||||||||||||||||||||||||||||||||||||||||||||||||||||||||||||||||||||||||.||.|||.||||||||||||-||||.||||||||||...|||||||||||.|||.|||||.||-|-||||||||||||||| ACCGTCTGGTTTTTATATGAAGTTCTTTCCTTCACTACCACAGGCCTCAAAGCGGTCCAAATCTCCACTTGCAGATTCTACAAAAAGAGTGATTCCAATCTGCTCTATCAATAGGATTGTTCAACTCCATGAGTTGAATGCCATCCTCACAAAGTC-GTTTCTGAGAATGCT<br>+<br>TCCGTTTGCCTTTTATATGAAGTTCCTTCCTGT-ACTACCGTAGGCCTCAAAGCAGTCCAAATCTCCATTTGCAGATTCTACAAAAAGAGTGATTCCAATCTGCTCTATCAATAGGATTGTTCAACTCCATGAGTTGAATGCCATCCTCACAAAGTCGTTTCTGAGAATGCT.||||.||..| ||||||||||||||.|||||-|-||||||..||||||||||||.|||||||||||||.||||||||||||||||||||||||||||||||||||||||||||||||||||||||||||||||||||||||||||||||||| ACCGTCTGGTTTTTATATGAAGTTCTTTCCT-TCACTACCACAGGCCTCAAAGCGGTCCAAATCTCCACTTGCAGATTCTACAAAAAGAGTGATTCCAATCTGCTCTATCAATAGGATTGTTCAACTCCATGAGTTGAATGCCATCCTCACAAAGTCGTTTCTGAGAATGCT			
P-D	A	4.7	10
TCCGTTTAGCTTTTAGGTGAAGATTATCCCGTTTCCAACGAAACCTTCAAAGAGGTCCAAATATCCCCTTGCGGATCCCACAGAAAGAGTGTTTCGAAACTGCTGTTTCAAA-GGAATCTTCAACTCTGTGAGTTGAATGCAATCATCACAAAGAAGTTTCTGACAATGCT<br>-|-|||||||||||||||||.||||||||.|||||||||||||||||||||.||.|||||||||||||||||||.|||||||||||||||||||||||||||||||||||-|||||||||||||||||||||||||||||||||||||||||||||||||||||||||| -C-GTTTAGCTTTTAGGTGACGATTATCCAGTTTCCAACGAAACCTTCAAATAGATCCAAATATCCCCTTGCGGTTCCCACAGAAAGAGTGTTTCGAAACTGCTGTTTCAAAAGGAATCTTCAACTCTGTGAGTTGAATGCAATCATCACAAAGAAGTTTCTGACAATGCT			
K-M(K-J/I-H)	G+I	4.09+19.88	8
TCTATCTAGTTTTTATGTGAAGATATTTCCTTTTCCACCACAGGCCTCAAAGCCCTCCAAACGTCCACTTGCAGATTCTCGAAAAAGAGTGTTTCATAGCTGCTCTTTCAAA-GGAAAGTTCAACTCTGGGAGTTGAATACAAACATCACAAAGTA-GTTTCCGAGAATGCT<br>||||||||||||||||||||||||||||||||||||||||||||||||||||||||||||||||||||||||||||||||||||||||||||||||||||||||||||||||-|||||||||||||||||||||||||||||||||||||||||.|-|||-|.|||..|||| TCTATCTAGTTTTTATGTGAAGATATTTCCTTTTCCACCACAGGCCTCAAAGCCCTCCAAACGTCCACTTGCAGATTCTCGAAAAAGAGTGTTTCATAGCTGCTCTTTCAAAAGGAAAGTTCAACTCTGGGAGTTGAATACAAACATCACAAAGAATGTT-CTGAGTTTGCT<br>+<br>TCTGTCTAGGTTTGATGTGAAGATATAGACGTTTCAAACGA-AGGC-TACAAAGTGGTCAAAATATACACTTGCAGATTCTACTACAAGG-GTGTTGCAAACCTGAACTATCAAA-GGAAGGTTCAACTCTGTGAGTTGAATACAAACATCACAAAGAATGTTCTGAGTTTGCT<br>|||.|||||.|||.||||||||||||...|.||||.|.|-|-||||-|-|||||...||.|||..|.||||||||||||-|.|.||.|-|||||.||.|.|||..||.|||||-||||.|||||||||||.||||||||||||||||||||||||||||||||||||||||| TCTATCTAGTTTTTATGTGAAGATATTTCCTTTTCCACC-ACAGGCCT-CAAAGCCCTCCAAACGTCCACTTGCAGATTCT-CGAAAAAGAGTGTTTCATAGCTGCTCTTTCAAAAGGAAAGTTCAACTCTGGGAGTTGAATACAAACATCACAAAGAATGTTCTGAGTTTGCT			
I-S	I	4.09	5
TCTGTCTAGGTTTGATGTGAAGATATAGACGTTTCAAACGAAGGCTACAAAGTGGTCAAAATATACACTTGCAGATTCTACTACAAGGGTGTTGCAAACCTGAACTATCAAAGGAAGGTTCAACTCTGTGAGTTGAATACAAACATCACAAAGAATGTTCTGAGTTTGCT-<br>|||||||||||||||||||||||||||||||||||||||||||||||||||||||||||||||||||||||||||||||||||||||||||.|||||||||.|||||||||||||||||||||||||||||.|||||.|||.|||||||||.|||||||||||||||||-<br>TCTGTCTAGGTTTGATGTGAAGATATAGACGTTTCAAACGAAGGCTACAAAGTGGTCAAAATATACACTTGCAGATTCTACTACAAGGGTGATGCAAACCTCAACTATCAAAGGAAGGTTCAACTCTGTGAGATGAATGCAACCATCACAAAAAATGTTCTGAGTTTGCTT			

H-V(H-G/A-L)	J+E	3.57+10.12	4

```
TCCGTTCAGTTATGGGAAGTTGATCCCGTTTCCAACGAAATCCTCAGAGAGGTCCAAATATCCCCTTGCAGATTCTACAAAACGTGTGTTTGGAAACTGCTCCATCATAACGAATGTTCAGCTCCCTGAGTTAAACTCCATCGTCACAAAGAATTTTCTGAGAGTGCT
-||||||||||||||||||||||||||||||||||||||||||||||||||||||||||||||||||||||||||||||||||||||||||||||||||||||||||||||||||||||||||.||||||.||||||||||.||||||||||.||||||||.||||
-CCGTTCAGTTATGGGAAGTTGATCCCGTTTCCAACGAAATCCTCAGAGAGGTCCAAATATCCCCTTGCAGATTCTACAAAACGTGTGTTTGGAAACTGCTCCATCATAACGAATGTTCAGCTCTCTGAGTGAAACTCCATCATCACAAAGAATATTCTGAGAATGCT
+
TGTGTTTAGTTATGGGAAATT-ATTCCCGTTTCCAACGAAATCCTCAGAGAGCTCCAAATATCCACCT-GCAGATTCTACCAAAA-GTGTATTTGGAAACTGCTCCATCAAAAGGCATGTTCAGCTCTGTGAGTGAAACTCCATCATCACAAAGAATATTCTGAGAATGCT
..-|||.|||||||||||.||-||-||||||||||||||||||||||||||.|||||||||||-|||-||||||||||-||||-||||.||||||||||||||||||||.||.|.|||||||||||.||||||||||||||||||||||||||||||||||||||||||||||||||||||
CC-GTTCAGTTATGGGAAGTTGAT-CCCGTTTCCAACGAAATCCTCAGAGAGGTCCAAATATCC-CCTTGCAGATTCTAC-AAAACGTGTGTTTGGAAACTGCTCCATCATAACGAATGTTCAGCTCTCTGAGTGAAACTCCATCATCACAAAGAATATTCTGAGAATGCT
```

rare monomers

W-S	B	9.14	3

```
TCTCTCTCGTCTTTCTGTGAAGATAAAGGAAAAGGCTTTCAGGCCTTTTCCACCACAGGCCTGAAAGCGCTCCAAATGTCCACTTGCAGATTCTGCCAAAAGAATATTTCAAAACTGCTCTATGAAAAGCAATGTTAAACTCTGTGGCTCGAACACAAACATCACAAAGCAGTTTCTGAGAAT
GCT
||||||||||||||.|||||||||-----------||||---|-|||||||||||||||||||||||||||||||||||.||||||||||||||||||||||||||||||||||||||||||||||||||||||||||||||||||||||||||||||||||||||||||||||||||||||||||
|||
TCTCTCTCGTCTTTATGTGAAGATA-----------TTTC---C-TTTTCCACCACAGGCCTGAAAGCGCTCCAAATGTCCNCTTGCAGATTCTGCCAAAAGAATATTTCAAAACTGCTCTATGAAAAGCAATGTTAAACTCTGTGGCTCGAACACAAACATCACAAAGCAGTTTCTGAGAAT
GCT
```

Q-S	B	12.9	3

```
TCTCTCTCGTCTTTCTGTGAAGATAAAGGAAAAGGCTTTCAGGCCTTTTCCACCACAGGCCTGAAAGCGCTCCAAATGTCCACTTGCAGATTCTGCCAAAAGAATATTTCAAAACTGCTCTATGAAAAGCAATGTTAAACTCTGTGGCTCGAACACAAACATCACAAAGCAGTTTCTGAGAAT
GCT
|||||||||||||||||||||||||||||||||||||||||||||||||.||||||||||---|||----|-----|--|---|-|------|||||||||||||||||||||||||||||||||||||||||||||||||||||||||||||||||||||||||||||||||||||||||||||||
|||
TCTCTCTCGTCTTTCTGTGAAGATAAAGGAAAAGGCTTTCAGGCCTTTGCCACCACAGGC---AAA----T-----T--C---T-GC-----CTGCCAAAAGAATATTTCAAAACTGCTCTATGAAAAGCAATGTTAAACTCTGTGGCTCGAACACAAACATCACAAAGCAGTTTCTGAGAAT
GCT
```

J-B(J-I/S-B)	H+C	9.35+6.43	2

```
TCTGTTTAGTTTTATGTGAAGATGATCCCGTTTCAGTGAAATCTTCAAAGAGGTCCACATATCCCCTTGCAGATTCCA-AAGAAAGAGGGTTTCAAAACTGCTCCATCAGAAGGATTGTTCAACTCTGTGAGTTGAATGCAGTCATCGCAGAAAACTTTCTGAGAATGCT
|||||||||||||||||||||||||||||||||||||||||||||||||||||||||||||||||||||||||||||||||.|-||-|||||..||||||||||||||||||||.|||||..||||||||.|||||.|||||||||.|||||.|..|.||.|||||||||||||
TCTGTTTAGTTTTATGTGAAGATGATCCCGTTTCAGTGAAATCTTCAAAGAGGTCCACATATCCCCTTGCAGATTCTACAA-AAAGACAGTTTCAAAACTGCTCCATCAAAAGGAGGGTTCAACTGTGTGACTTGAATGCAATCATCACTCAGAAGTTTCTGAGAATGCT
+
TCAGTTTAGTTTTCTGTGGAA-AT-ATTCCCGTTTCCAAAGAAATCTTCAAAGAGGTCCACGTATCCACTTACAGATTCTACAAAAAGACAGTTTCAAAACTGCTCCATCAAAAGGAGGGTTCAACTGTGTGACTTGAATGCAATCATCACTCAGAAGTTTCTGAGAATGCT
||.|||||||||.||||-||-||-||-|||||||||..|||||||||||||||||||||.|||||.|||.|||||||||||||||||||||||||||||||||||||||||||||||||||||||||||||||||||||||||||||||||||||||||||||||||||||||||||||
TCTGTTTAGTTTTATGTG-AAGATGAT-CCCGTTTCAGTGAAATCTTCAAAGAGGTCCACATATCCCCTTGCAGATTCTACAAAAAGACAGTTTCAAAACTGCTCCATCAAAAGGAGGGTTCAACTGTGTGACTTGAATGCAATCATCACTCAGAAGTTTCTGAGAATGCT
```

B-T	D	3.5	

```
TCTCTTTAGTTTTACGTGAACATATACCCGTTTCGAACGAAGGCCACCCAGTGGTCCAAATATCCACTTGCAGATTCTACAGAAAGAGTGTTTCGAACCTGAACTCTCAAAGGCAGGTTCATCTCTGCGAGTTAAATGCATTCATCATGAAGAACTTTCTCAGAGTGTT-
|||||||||||||||||||||||||||||||||||||||||||||||||||||||||||||||||||||||||||||||||||||||||.||||||||||||||||||||||||||||.||||||||||||.|||||||||.||||||||||||||||||.||||||-
TCTCTTTAGTTTTACGTGAACATATACCCGTTTCGAACGAAGGCCACCCAGTGGTCCAAATATCCACTTGCAGATTCTACAGAAAGAGTTTTTCGAACCTGAACTCTCAAAGGCAGGTTGATCTCTGCGAGTTCAATGCATTCAACATGAAGAACTTTCTCAGCGTGTTT
```

T-L	E	4.7	

```
TGTGTTTAGTTATGGGAAATTATT-CCCGTTTCCAACGAAATCCTCAGAGAGCTCCAAATATCCACCTGCAGATTCTACCAAAAGTGTATTTGGAAACTGCTCCATCAAAAGGCATGTTCAGCTCTGTGAGTGAAACTCCATCATCACAAAGAATATTCTGAGAATGCT
-||||.||||||.||||||||||-|||-|||||||.||||||||||||||.|||||||||||||||.||||||||||||||||||||||||||||||||||||||||||||||||||||||||||||||||||||||||||||||||||||||||||||||||||||||||||||
-GTGTATAGTTAAGGGAAATTATTGCCC-TTTCCAATGAAATCCTCAGAGAGGTCCAAATATCCACCTGTAGATTCTACCAAAAGTGTATTTGGAAACTGCTCCATCAAAAGGCATGTTCAGCTCTGTGAGTGAAACTCCATCATCACAAAGAATATTCTGAGAATGCT
```

Y-F	K	3.5	

```
ACCGTCTGGTTTTTATATGAAGTTCTTTCCTTCACTACCACAGGCCTCAAAGCGGTCCAAATCTCCACTTGCAGATTCTACAAAAAGAGTGTTTGCAAACTGCTCTATCAAA-GGAATGTTCAACTCTGGGAGTTGAATGCAATCATCACAGAGCAGTTTCTGAGAATGCT
|||.|||.||||||||||||||||||||||||.||.|||||||||||||||||||||.|||||||||||||||||||||||||||||||||||||||||||||||||||||-||||||||||||||||||||||||||||||||||||||||||||||||||||||||||||||||||
ACCATCTAGTTTTTATATGAAGTTCTTTCCTTTACGACCACAGGCCTCAAAGCGGTCTAAATCTCCACTTGCAGATTCTACAAAAAGAGTGTTTGCAAACTGCTCTATCAAAAGGAATGTTCAACTCTGGGAGTTGAATGCAATCATCACAGAGCAGTTTCTGAGAATGCT
```

H-L(H-G/A-L)	J+E	7.7+5.95	

```
TCCGTTCAGTTATGGGAAGTTGATCCCGTTTCCAACGAAATCCTCAGAGAGGTCCAAATATCCCCTTGCAGATTCTAC-AAAACGTGTGTTTGGAAACTGCTCCATCATAACGAATGTTCAGCTCCCTGAGTTAAACTCCATCGTCACAAAGAATTTTCTGAGAGTGCT
-|||||||||||||||||||||||||||||||||||||||||||||||||||||||||||||||||||||||||||||||-||||-||||.|||||||||||||||||||.||.|.|||||||||||..|||||.||||||||||.||||||||||.||||||||.||||
-CCGTTCAGTTATGGGAAGTTGATCCCGTTTCCAACGAAATCCTCAGAGAGGTCCAAATATCCCCTTGCAGATTCTACCAAAA-GTGTATTTGGAAACTGCTCCATCAAAAGGCATGTTCAGCTCTGTGAGTGAAACTCCATCATCACAAAGAATATTCTGAGAATGCT
+
TGTGTTTAGTTATGGGAAATT-ATTCCCGTTTCCAACGAAATCCTCAGAGAGCTCCAAATATCCACCT-GCAGATTCTACCAAAAGTGTATTTGGAAACTGCTCCATCAAAAGGCATGTTCAGCTCTGTGAGTGAAACTCCATCATCACAAAGAATATTCTGAGAATGCT..-|||.||||||
|||||.||-||-|||||||||||||||||||||||||||.|||||||||||-|||-||||||||||||||||||||||||||||||||||||||||||||||||||||||||||||||||||||||||||||||||||||||||||||||||||||||
CC-GTTCAGTTATGGGAAGTTGAT-CCCGTTTCCAACGAAATCCTCAGAGAGGTCCAAATATCC-CCTTGCAGATTCTACCAAAAGTGTATTTGGAAACTGCTCCATCAAAAGGCATGTTCAGCTCTGTGAGTGAAACTCCATCATCACAAAGAATATTCTGAGAATGCT
```

R-C(E-D/D-C)	A+B	15.88+20.43	1

```
TCCGTTTAGCTTTTAGGTGAAGATTATCCCGTTTCCAACGAAACCTTCAAAGAGGTCCAAATATCCCCTTGCGGATCCCAC-AGAAAGAGTGTTTCGAAACTGCTGTTTCAAAGG-AATCTTCAACTCTGTGAGTT-GAATGCAATCATCACAAAGAAGTTTCTGACAATGCT
-|-||||||||||.||-||||||||||||||||||||||||.||||||||||||||||||||||||||||||||||||||||..|-|-|||||.|.||||.||||||||.|.|.|||.|-|||.||.|||||||||-|.|-|||..|||.||||||||||.|||||||||.||||||
-C-GTTTAGCTTTAAG-TGAAGATTATCCCGTTTCCAACGAAATCTTCAAAGAGGTCCAAATATCCCCTTGCGGATCCTGCCA-AAAGAATATTTCAAAACTGCTCTATGAAAAGCAATGTTAAACTCTGTG-GCTCGAACACAAACATCACAAAGCAGTTTCTGAGAATGCT
+
TC-TCTCTCGTCTTTCTGTGAAGATAAAGGAAAAGGCTTTCAGGCCTTTTCCACC-ACAGGCCTGAAAGCGCTCCAAATGTCCACTTGCAGATTCTGCCAAAAGAATATTTCAAAACTGCTCTATGAAAAGCAATGTTAAACTCTGTGGCTCGAACACAAACATCACAAAGCAGTTTCTGAGA
ATGCT
-|-|-|-|.|-||||..||||||||.|----------|--|---||.||||||.|-|.|.-|.|.||||.|.||||||||.|||.|||||.|||.|||||||||||||||||||||||||||||||||||||||||||||||||||||||||||||||||||||||||||||||||||||||||||
|||||
-CGT-T-TAG-CTTTAAGTGAAGATTA----------T--C---CCGTTTCCAACGAAAT-CTTCAAAGAGGTCCAAATATCCCCTTGCGGATCCTGCCAAAAGAATATTTCAAAACTGCTCTATGAAAAGCAATGTTAAACTCTGTGGCTCGAACACAAACATCACAAAGCAGTTTCTGAGA
ATGCT
```

G-E(G-F/F-E)	K+L	10.52+14.6	1

```
ACCGTCTGGTTTTTATATGAAGTTCTTTCCTTCACTACCACAGGCCTCAAAGCGGTCCAAATCTCCACTTGCAGATTCTACAAAAAGAGTGTTTGCAAA-CTGCTCTATCAAAGG-AATGTTCAACTCTGGGAGTTGAATGCAATCATCACAGAGCAGTTTCTGAGAATGCT--
|||||||||||||||||||||||||||||||||||||||||||||||||||||||||||||||||||||||||||||||||.||.||||||.||||---||||-||||.|||||||||-||.|||||||||||.|||.||||||||||.||||||.||.||.||||||||||||--
ACCGTCTGGTTTTTATATGAAGTTCTTTCCTTCACTACCACAGGCCTCAAAGCGGTCCAAATCTCCACTTGCAGATTGTAGAAAAAAGTGTGT---CAAAGCTGCGCTATCAAAGGGAAAGTTCAACTCTGTGAGGTGAATGCAAACATCCCAAAGAAGTTTCTGAGAATGCTTC
+
```

<table>
<tr><td colspan="4">TCTATGTCGTTTTTAGGA-GAAGATATTTCCTTTTCCAACACAGTCCTCCAAGCCCG-CTAAATAGCCACTTGCACATTGTAGAAAAAGTGTGTCAAAGCTGCGCTATCAAAGGGAAAGTTCAACTCTGTGAGGTGAATGCAAACATCCCAAAGAAGTTTCTGAGAATGCT--
.|..|.|.|||||||.-|-||||.|.|||||||..|.|.|||||.||||.||||.-|-|.||||..|||||||||.|||||||||||||||||||||||||||||||||||||||||||||||||||||||||||||||||||||||||||||||||||||||||||||||||||--
ACCGTCTGGTTTTTAT-ATGAAGTTCTTTCCTTCACTACCACAGGCCTCAAAGCG-GTCCAAATCTCCACTTGCAGATTGTAGAAAAAGTGTGTCAAAGCTGCGCTATCAAAGGGAAAGTTCAACTCTGTGAGGTGAATGCAAACATCCCAAAGAAGTTTCTGAGAATGCTTC</td></tr>
<tr><td>A-F(A-L/G-F)</td><td>E+K</td><td>10.71+15.88</td><td>1</td></tr>
<tr><td colspan="4">TGTGTTTAGTTATGGGAAATTATTCCCGTTTCCAACGAAATCCTCAGAGAGCTCCAAATATCCACCTGCAGATTCTACCAAAAGTGTATTTGGAAACTGCTCCATCAAAAGGCATGTTCAGCTCTGTGAGT-GAAACTCCA-TCATCACAAAGAA-TATTCTGAGAATGCT
-|||||||||||||||||||||||||||||||||||||||||||||||||||||||||||||||||||||||||||||.|||||.||.||||.|||||||||.|||||||||.|||||||.|||||.||||-|||--|.||-||||||||.||.|-|-|||||||||||||
-GTGTTTAGTTATGGGAAATTATTCCCGTTTCCAACGAAATCCTCAGAGAGCTCCAAATATCCACCTGCAGATTCTACAAAAAGAGTGTTTGCAAACTGCTCTATCAAAAGGAATGTTCAACTCTGGGAGTTGAA--TGCAATCATCACAGAGCAGT-TTCTGAGAATGCT
+
ACCGTCTGGTTTTTATATGAAGTTCTTTCC-TT-CACTACCACAGGCCTCAAAGCGGTCCAAATCTCCACTTGCAGATTCTACAAAAAGAGTGTTTGCAAACTGCTCTATCAAA-GGAATGTTCAACTCTGGGAGTTGAATGCAATCATCACAGAGCAGTTTCTGAGAATGCT
..-||.|.|||---||..|||.||.||.||-||-|-|.||.|.|.-|||||.||.|.|||||||.|||||.|||||||||||||||||||||||||||||||||||||||||||-||||||||||||||||||||||||||||||||||||||||||||||||||||||||||
GT-GTTTAGTT---ATGGGAAATTATTCCCGTTTC-CAACGAAAT-CCTCAGAGAGCTCCAAATATCCACCTGCAGATTCTACAAAAAGAGTGTTTGCAAACTGCTCTATCAAAAGGAATGTTCAACTCTGGGAGTTGAATGCAATCATCACAGAGCAGTTTCTGAGAATGCT</td></tr>
<tr><td>B-H(B-A/I-H)</td><td>D+I</td><td>12.87+8.77</td><td>1</td></tr>
<tr><td colspan="4">TCTCTTTAGTTTTTACGTGAACATATACCCGTTTCGAACGAAGGCCACCCAGTGGTCCAAATATCCACTTGCAGATTCTAC-AGAAAGAGTGTTTCGAACCTGAACTCTCAAAGGCAGGTTCATCTCTGCGAGTTAAATGCATTCATCATGAAGAACT-TTCTCAGAGTG-TT
|||||||||||||||||||||||||||||||||||||||||||||||||||||||||||||||||||||||||||||||||-|.||.|-|||||.|.||||||||||.|||||||.|||||||.|||||.|||||.|||.||..|||||..|||||-|-||||.||..||-||
TCTCTTTAGTTTTTACGTGAACATATACCCGTTTCGAACGAAGGCCACCCAGTGGTCCAAATATCCACTTGCAGATTCTACTACAAGG-GTGTTGCAAACCTGAACTATCAAAGGAAGGTTCAACTCTGTGAGTTGAATACAAACATCACAAAGAA-TGTTCTGAGTTTGCTT
+
TCTGTCTAGGTTTGATGTGAAGATATAGACGTTTCAAACGAAGGCTACAAAGTGGTCAAAATATACACTTGCAGATTCTACTACAAGGGTGTTGCAAACCTGAACTATCAAAGGAAGGTTCAACTCTGTGAGTTGAATACAAACATCACAAAGAATGTTCTGAGTTTGCT-
|||.|.|||.|||.|.|||||.|||||..||||||.|||||||||.||..|||||||.||||||.|||||||||||||||||||||||||||||||||||||||||||||||||||||||||||||||||||||||||||||||||||||||||||||||||||||||-
TCTCTTTAGTTTTTACGTGAACATATACCCGTTTCGAACGAAGGCCACCCAGTGGTCCAAATATCCACTTGCAGATTCTACTACAAGGGTGTTGCAAACCTGAACTATCAAAGGAAGGTTCAACTCTGTGAGTTGAATACAAACATCACAAAGAATGTTCTGAGTTTGCTT</td></tr>
</table>

Table MonomersComparison. Comparing the (shifted) monomer-set generated by MonomerGenerator with the reference monomers from cenX. The first column corresponds to the identifier of the generated monomer after shift. Monomer identifier X-Y represents the shift of monomers X and Y from the initially generated monomer-set. The second column corresponds to the closest reference monomer. Two monomer names separated by "+" refer to two monomers that form a hybrid monomer. For example, "G-K" is a hybrid of monomer K and F (Dvorkina et al., 2020). The third column is the divergence with the closest reference monomer (or a pair of the divergence values with two original monomers if the generated monomer represents a hybrid). The fourth column is the number of *M*-blocks in cenX that correspond to the generated monomer *M*. For each generated monomer, the table shows the global alignment to the closest reference monomer (the mismatch and indel penalties are 1, and the match premium is 1). The first (second) line in the alignment corresponds to the reference (generated) monomer. In the case of hybrid monomers, segments corresponding to each reference monomer are highlighted.

Supplementary Note: Pseudocode and complexity analysis of MonomerGenerator and HORDecomposer

```
MonomerGenerator
Input: A string-set Centromeres, a string InitialMonomer, an integer
maxResolvedDivergence
Output: A string-set Monomers

Monomers <- a string-set consisting of a single string InitialMonomer

while Monomers do not resolve Centromeres {
       # Launch StringDecomposer to generate all blocks in Centromeres
       AllBlocks <- Blocks(Centromeres, Monomers)
       for each monomer M in Monomers {
       # identify resolved M-blocks in the set MBlocks
       MBlocks <- ResolvedBlocks(AllBlocks, M, maxResolvedDivergence)
       if MBlocks is empty {
                    remove monomer M from Monomers
       } else {
                    # update consensus for monomer M
             M <- Consensus(MBlocks)
             }
       }

   # identify blocks that are not resolved by any monomers
```

```
    UBlocks <- UnresolvedBlocks(AllBlocks, Monomers, maxResolvedDivergence)

    # create a block graph where vertices correspond to unresolved blocks
       # and edges connect similar unresolved blocks
    BlockGraph <- graph with vertex-set UBlocks and empty edge-set
       for each pair of blocks v and w in UBlocks:
             if EditDistance(v, w) < maxResolvedDivergence / 2
                   add edge between v and w to BlockGraph
    Component <- a maximum-size connected component in BlockGraph
    NewMonomer <- consensus of all blocks in Component
    add NewMonomer to MonomerSet
}
return MonomerSet
```

The time complexity of each iteration of the while loop is dominated by two factors: running StringDecomposer and identification of the largest connected component in the block-graph.

Running time of String Decomposer. The running time of StringDecomposer is $O(length(Centromeres) * length(Monomers))$, where *length*(*Strings*) is the total length of all strings in string-set *Strings* (dvorkina et al., 2020). For the human genome, *length(Centromeres)* ~ 70 Mbp, *length(Monomers)* ~ 400 * 171 = 68,400bp (at the final stage).

Block-graph. Since the vertices of the graph are the (monomeric) blocks and the monomers have a rather conserved length |*Monomer*|, the complexity of constructing the block-graph is $O(|MBlocks|^2 * |Monomer|^2)$. For the human genome, |*MBlocks*| ~ 400,000, |*Monomer*| ~ 171bp.

The worst-case number of iterations is |*Monomers*| ~ 400. Thus, the complexity of a naive implementation of MonomerGenerator is

$$O(|Monomers| * (length(Centromeres) * length(Monomers)) + |MBlocks|^2 * |Monomer|^2)).$$

Supplementary Note “Constructing connected components of the block-graph” describes a faster algorithm for constructing the connected components of the block-graph.

```
HORDecomposer
Input: A string Monocentromere over the alphabet Monomers, and integers
MinCount, MaxLength, and MinWeight
Output: A string-set HORs over the alphabet Monomers and a string-set
HORCentromere over the HORs alphabet

HORCentromere <- Monocentromere
HORs <- empty string-set
HORnumber <- 0
while True {
      # Find a new heaviest potential HOR in HORCentromere
      HOR, HORWeight <- None, 0
      for each non-trivial substring S in HORCentromere {
      if count(HORCentromere, S) > MinCount
```

```
        if length(S) < MaxLength
              if weight(HORCentromere, S) > MinWeight
              NewHORWeight <- weight(HORCentromere, S)
                          if NewHORWeight > HORWeight
                    HOR, HORWeight <- S, NewHORWeight
        }
    # Check if a new HOR was identified
        if HOR is None
              return HORs, HORCentromere
        add HOR to HORs
        HORnumber <- HORnumber+1
    replace all occurrences of HOR in HORCentromere with a symbol HORnumber
}
```

At each iteration of the while loop, HORDecomposer considers all short substrings of *HORCentromere*. Their total number is |*HORCentromere*| * *MaxLength*. The running time of the naive algorithm for computing the *weight* function is O(|*HORCentromere*|). The number of iterations of the while loop is O(|*HORs*|). Thus, the running time of HORDecomposer is $O(|HORs|\ |HORCentromere|^2 * MaxLength)$.

Supplementary Note: Identifying non-monomeric regions

Limitations of StringDecomposer. Given an arbitrary set of strings *Monomers* and a string *S* as an Input, the String Decomposition Problem is to find an optimal decomposition of *S* into strings from *Monomers* (Dvorkina et al., 2020). However, the challenge of properly defining the set of input monomers remained outside the scope of the String Decomposition Problem. In the case of centromeres, the monomer-set can be defined as the set of all known human monomers (or, in the case of a specific centromere, the set of monomers that form this centromere). However, some monomers are still unknown and some centromeres contain non-monomeric regions, such as LINE elements, Alu repeats, and other transposons. CenromereDecomposer — an extension of StringDecomposer — identifies non-monomeric regions in centromeres, adds these non-monomeric regions as additional *pseudo-monomers* to the initial monomer-set, and generates a new string decomposition that takes into account these pseudo-monomers.

Non-monomeric regions. Given a centromere and a set of monomers *Monomers,* the decomposition of this centromere into monomers is represented as a *monocentromere* $M_1, \ldots, M_K$, where M_i stands for a monomer. For each monomer *M* in a monocentromere, we define *identity(M), start(M),* and *end(M)* as its sequence identity, its starting position in the centromere, and its ending position in the centromere, respectively.

A monomer *M* in a monocentromere is called *weak* if *identity(M)* < *MinMonomerIdentity* (the default values *MinMonomerIdentity*=85%). A substring of a monocentromere is called *weak* if all its monomers are weak but its flanking monomers are not weak. Each substring $M_i, \ldots, M_j$ of a monocentromere defines a *submonocentromere* that begins at $start(M_i)$ and ends at $end(M_j)$. We refer to the weak substrings of a monocentromere as *non-monomeric substrings*.

When a monomeric region (like a LINE repeat) is inserted into a centromere at some position in a monomer *M*, it breaks this monomer into a prefix (located before the inserted region) and suffix (located after the inserted region). Therefore, the extracted non-monomeric substring typically represents not only the actual *pure non-monomeric region* (such as the inserted LINE element), but also the prefix and suffix of the monomers broken by this insertion. In order to extract the *pure non-monomeric region*, CentromereDecomposer trims each non-monomeric substring of length greater than *MonomerLength* (*MonomerLength* = 171bp) as described below.

Trimming non-monomeric regions. For each monomer *M* from the set *Monomers,* CentromereDecomposer computes the global alignment against the non-monomeric substring S. It further finds the maximum score in the alignment matrix (referred to as *score*(*M,S*)) that defines the *prefix alignment* between a prefix of the monomer (referred to as $prefix_S(M)$) and a prefix of the non-monomeric substring (referred to as $prefix_M(S)$). It further selects a monomer *M* maximizing *score*(*M,S*)) among all monomers and trims the non-monomeric string by deleting $prefix_M(S)$. Similarly, centromereDecomposer defines $suffix_M(S)$ and trims the non-monomeric string by deleting $suffix_M(S)$. The resulting trimmed string is referred to as a *pure non-monomeric string.*

Clustering non-monomeric substrings. CentromereDecomposer extracts all non-monomeric substrings from the centromere, transforms them into pure non-monomeric substrings, combines them into the set *NonMonoStrings,* and clusters substrings in this set using single-linkage clustering. Two strings *S* and *S'* from *NonMonoString* are clustered together if $distance(S,S')/\min(|S|,|S'|) < MaxDiff$, where *distance*(*S,S'*) stands for the edit distance between *S* and *S'* (the default values for *MaxDiff*=0.05). Each resulting *non-trivial* cluster (i.e., a cluster containing more than one element) contains similar non-monomeric substrings that may represent multiple similar regions of a centromere. For each non-trivial cluster, CentromereDecomposer identifies a cluster representative as a sequence with the smallest total edit distance to other sequences in this cluster. The set of all such representatives describes all non-monomeric regions in a centromere.

For each short non-monomeric cluster representative with length <= *MonomerLength* (*MonomerLength*=200bp), CentromereDecomposer checks if its sequence represents a *corrupted* (e.g., highly diverged) monomer rather than a real non-monomeric sequence. For each such representative, we find the best fitting alignment between its sequence *RepresentativeString* and each monomer in the monomer-set. CentromereDecomposer classifies a cluster as a *corrupted monomer cluster* if the minimal edit distance between *RepresentativeString* is below 0.3*|*RepresentativeString*| (or below 20 in the case of very short representative strings of length less than 70 nt).

The method described above can be applied to both the centromere and each read in a read-set as described in Supplementary Note "Non-monomeric regions in human centromeres." In the case of error-prone reads, we launch CentromereDecomposer with different parameters to account for high error rates.

Supplementary Note: Non-monomeric regions in human centromeres

We launched CentromereDecomposer on the T2T human genome assembly as well as on HiFi and ONT reads from cenX and cen8 (https://github.com/nanopore-wgs-consortium/CHM13) with reference monomers (we ignored cen6 in this analysis since it doesn't contain non-monomeric regions). We recruited 7337 and 4296 HiFi reads (2680 and 2943 ONT reads) to cenX and cen8, respectively (Bzikadze and Pevzner, 2020). Assemblies of cenX and cen8 were obtained from Bzikadze and Pevzner, 2020 and Logsdon et al. 2020, respectively. Previous studies revealed that there is a single LINE element in cenX (length ~6 kbp) and two Alu elements (length ~300 bp) at the beginning of cen8.

Table "NonMonomericCentromere" illustrates that CentromereDecomposer identified the LINE element in cenX and the Alu elements in cen8. In addition, it has also found a "shortened" partial monomer of length 113 that occurs three times in cen8 assembly. Table NonMonomericReads illustrates CentromereDecomposer results on HiFi (8 non-monomeric reads for cenX and 135 non-monomeric reads for cen8) and ONT reads. In cenX, only one cluster contains more than a single non-monomeric sequence and the representative of this particular cluster is classified as a LINE1 element by RepeatMasker. In cen8, there are three clusters: the first cluster represents Alu repeat and has representative length ~300bp, the second cluster represents a corrupted monomer (similar to monomer B and O) of length 113 bp, that occurs three times in assembly), the third cluster represents another corrupted monomer that represents a ~122bp suffix of monomer A. It was identified as a reliable monomer in assembly albeit with a low identity 79%.

Although the number of clusters identified through analysis of ONT reads has increased in comparison to the accurate HiFi reads, CentromereDecomposer generated roughly the same non-monomeric blocks with a large number of representatives but also resulted in 19 small clusters (size < 4) that represent corrupted monomers with lengths < 171bp. In cen8, in addition to the original Alu cluster and two corrupted monomer clusters, CentromereDecomposer found a cluster representing a segment of LINE1 element (length of the segment ~2500bp), which occurs in the pericentromere of cen8.

	**# non-monomeric strings**	**# clusters**	**cluster sizes**	**RepeatMasker classification**
cenX	1	1	|C1|=1	LINE element
cen8	2	2	|C1|=2<br>|C2|=3	Cluster 1 represents Alu repeat<br>Cluster 2 represents a corrupted monomer of length 113 bp that occurs three times in the centromere

Table NonMonomericCentromere. CentromereDecomposer results on assemblies of cenX and cen8. cenX contains a single LINE element. cen8 contains two non-monomeric insertions: two Alu repeats, and a corrupted monomer that occurs three times.

	# non-monomeric reads	# non-monomeric strings	# clusters	# non-trivial clusters	cluster sizes	RepeatMasker classification
cenX	8	8	5	1	|C1|=3	LINE element
cen8	105	135	5	3	|C1|=14<br>|C2|=102<br>|C3|=17	Cluster 1 contains ALU repeat.<br>Cluster 2 contains a corrupted monomer of length 113 bp that occurs three times in the centromere (between monomers N and A or O and A).<br>Cluster 3 contains a corrupted monomer of length 122bp that occurs in the centromere with identity 79%

	# non-monomeric reads	# non-monomeric strings	# clusters	#non-trivial clusters	cluster sizes	RepeatMasker classification
cenX	264	535	502	7	|C1|=24<br>all other clusters have size at most 5	Cluster 1 contains LINE element<br>All other clusters represent corrupted monomers
cen8	507	1142	860	24	|C1|=129<br>|C2|=76<br>|C3|=28<br>|C4|=4<br>all other clusters have size at most 5	Cluster 1 contains ALU repeat<br>Cluster 2 contains a corrupted monomer of length 113 bp that occurs three times in assembly.<br>Cluster 3 contains a corrupted monomer of length 122 bp that appears once in assembly with identity 79%.<br>Cluster 4 contains a part of LINE1 element.<br>One of the remaining small clusters (of size 2) also represents an ALU repeat, but with severely corrupted monomer on both sides.<br>Representatives in all other clusters are short (<171bp)

Table NonMonomericReads. CentromereDecomposer results on HiFi (top) and ONT (bottom) reads from cenX and cen8. A read is called *non-monomeric* if it contains a non-monomeric string. There are 8 non-monomeric HiFi reads for cenX and 135 non-monomeric HiFi reads for cen8. CentromereDecomposer was launched with parameters *MaxDiff* = 0.05, *MinMonomerIdentity*=85% on HiFi reads and with parameters *MaxDiff* = 0.2, *MinMonomerIdentity*=70% on ONT reads. The number of non-trivial clusters was defined with the parameter *MinCoverage* = 2. Cluster 2 for cen8 contains a corrupted monomer of length 113 bp that occurs three times either between monomers N and A or monomers O and A. Cluster 3 for cen8 contains a corrupted monomer of length 122 bp that occurs one time between monomers O and C.

Supplementary Note: HOR and superHOR decomposition of cen6 and cen8

Supplementary Tables cen6decomposition and cen8decomposition provide information about HOR and superHOR decomposition of cen6 and cen8.

HOR name	HOR length	HOR	Count	Weight	Run-length
a	14	RSABCDEFGHIJKL	936	12168	4145
b	18	aMNOP	649	3067	1078
c	15	aT	279	859	690
d	16	RSABCDEFGHJIKNOP	11	759	519
e	11	SABCDEFGHIJ	6	646	459
f	15	aM	7	593	452
g	20	OPb	6	593	445

HOR decomposition	DEFGHIHKHKLRAgb[43]QeEFGHIJKNgb[2]db[2]dbd[3]b[6]QeEFGHIJKNgb[4]d[3]b[8]d[3]b[44]c[2]bcb[15]c[2]b[2]c[2]bc[2]bcb[11]fNeKLMNgb[4]cb[20]cbcbcbcbc[3]b[3]cbc[2]aOPcb[3]c[3]b[2]cb[3]cb[3]cbcb[3]QKLScb[5]cb[12]cb[2]cb[12]fNeKLScbc[2]b[2]c[3]bcbc[2]bc[3]b[2]c[8]bc[3]b[2]cQRABCDEFGHOPcbc[3]bc[5]bc[3]bc[2]bcb[2]c[3]fb[3]c[2]bc[2]b[3]c[2]b[2]c[2]bcb[2]cb[2]cb[2]cb[2]c[4]b[4]c[3]b[2]c[2]bcbcb[2]cbc[2]bc[2]bcbcbc[2]bcb[2]cbcb[22]c[11]bc[4]b[2]c[3]bc[2]bh3[3]b[2]c[10]bc[3]b[4]cbcb[2]c[2]fb[3]c[2]bcb[2]cb[2]c[2]b[2]cb[3]c[3]bc[2]bcbcb[2]cbc[2]bcbc[3]bcbh3bcbcbcbcb[6]c[2]bcbcbcb[7]c[3]bc[4]b[3]cb[4]cb[4]cbcb[19]cbcb[4]cb[2]cb[5]cb[6]c[2]bcb[5]cbcb[23]cbcb[4]cbcb[2]cbcb[8]cbcb[4]cb[2]cb[5]cb[6]cbcb[5]cbcb[24]cbcb[4]cbcb[3]cbcb[2]cbcbcb[4]fNeKLMNgcbcb[2]c[2]b[3]cb[23]cb[6]cbcb[6]cbcb[3]cb[3]c[2]bcbcb[3]cbcbfNeKA2MNOPc[4]b[4]cbcb[6]cb[3]cb[3]c[2]Qgb[14]cbcbcb[2]cb[5]cbcb[2]cb[8]c[4]b[4]c[2]b[5]c[4]bcb[3]c[4]bcb[2]cbcbfNO
superHOR decomposition	DEFGHIHKLSAgbReEFGHIJKNgbdbdbdbReEFGHIJKNgbdbdbcbfNeKLMNgbcbcaOPcbRKLTcbfNeKLTcbcRSABCDEFGHOPcbcfbcbfNeKLMNgcbfNeKLMNOPcbcRgbcbfNO

Supplementary Table cen6decomposition. Seven HORs in cen6 (top), HOR decomposition of cen6 into these HORs and monomers (middle), and superHOR decomposition of cen6 into eleven superHORs (bottom). cen6 is represented in the alphabet of 16 monomers. Different colors represent different superHORs. The length of the run-length encoding of the HOR (superHOR) sequence for cen6 is 445 (37).

HOR name	HOR length	HOR	Count	Weight	Run-length
a	7	ABCDEFG	907	5796	6454
b	8	ABCLHIJK	583	4704	2104
c	11	aHIJK	261	1734	993
d	6	ABCEFG	13	756	927
e	4	HIJK	6	709	909
f	3	ABC	8	707	893

HOR decomposition	DEFGec[16]fD_NM_EFGec[2]AKc[10]fD_NM_EFGef_NM_EFGecAKc[16]abacb[2]aba[2]ba[2]baba[2]ba[2]b[2]a[2]b[2]ab[2]c[6]abcbababa[3]ba[2]b[2]cbababab[2]c[2]a[3]babc[2]abc[3]ab[10]A_NM_BCLeb[2]A_NM_BCLeb[10]cb[7]c[2]aJKda[2]bab[2]ab[2]cabcabcabcab[2]cab[6]cbcb[2]cb[2]cabab[2]abababcababc[2]ababababababab[2]abcababababab[2]abab[2]ababab[2]abab[3]ab[3]abab[17]aba[2]bcababab[2]ababab[4]ca[3]dab[2]ab[5]ab[3]ab[13]ab[3]ab[7]ab[6]ab[5]aba[2]ba[2]baHBCDEFGb[3]a[2]b[3]ab[3]abab[2]ab[2]ab[2]abababababababababababababababab[2]ab[3]abafGbabababafG

	bab[3]ab[2]ab[2]ab[2]ab[4]abababab[2]ab[2]ababababababfIJKa<br>babababababababababababababab[2]ab[3]ab[3]ab[3]ababab[2]a<br>babababab[6]abababab[2]abababab[3]abab[3]abab[2]ab[3]aba[4]<br>b[5]aba[4]b[3]abababa[2]bababab[2]abab[5]abab[2]abab[2]abab<br>[2]ababab[2]abab[2]ababab[2]abab[3]ababa[2]c[2]ABFGa[6]c[2]<br>a[2]ca[8]dcaca[9]da[6]da[3]da[2]cac[2]acac[2]acacadacacacac<br>[2]acacadcaca[3]da[2]cacacac[2]acacadca[3]dacac[2]a[2]ca[98<br>]caca[2]b[2]a[2]ca[3]b[2]a[3]ca[2]ca[3]b[2]a[3]caca[3]ba[5]<br>ba[4]ba[26]d[2]a[4]bab[3]a[3]ba[7]bab[2]a[4]b[2]a[5]ba[2]ba<br>b[4]ab[3]ab[3]a[2]b[5]a[2]bacbacab[2]ab[3]a[2]b[3]a[2]bacab<br>[3]a[2]b[3]a[2]bacbacacab[3]a[2]b[3]acab[3]a[2]b[2]a[2]ca[2<br>]cbacba[2]cab[3]ab[3]a[2]b[2]a[2]b[3]a[2]b[4]a[2]b[2]a[2]b[<br>2]a[2]b[2]a[2]b[3]cbacbac[2]ab[2]acbac[2]baba[2]ba[2]b[4]ac<br>bac[2]a[2]b[2]ac[2]a[2]ba[2]b[2]a[2]ba[2]ba[2]b[4]acacababa<br>cbacac[2]baba[2]ba[2]b[4]acbac[2]a[2]b[2]ac[2]a[2]ba[2]b[2]<br>a[2]ba[2]ba[2]b[4]acacaba[2]ba[2]ba[2]ba[2]b[4]ac[2]a[2]ba[<br>2]ba[2]ba[2]b[4]acacaba[2]ba[2]ba[2]ba[2]b[4]ac[2]a[2]ba[2]<br>ba[2]ba[2]b[2]a[2]ba[2]ba[2]b[4]acbacacacacacbacbcbacbacb[5<br>]acabab[2]abfDE_NM_baca[2]b[6]cb[6]cb[3]cb[4]cb[3]c[4]bc[3]<br>abc[27]fDIJKc[47]aHIJBJKAB
superHOR decomposition	**DEFGecfD_NM_EFGecAKcfD_NM_EFGef_NM_EFGecAKcabacbabcbabcba<br>bcabA_NM_BCLebA_NM_BCLebcaJKdabcbcabcadabaHBCDEFGbabafGba<br>bafGbabfIJKabacABFGacadcacadadadacadcacadcadacabacabadaba<br>cbacabcbacabacbacbabacbacbabacbacbcbacabfDE_NM_bacabcbcab<br>cfDIJKcaHIJBJKAB**

Supplementary Table cen8decomposition. Six HORs in cen8 (top), HOR decomposition of cen6 into these HORs and monomers (middle), and superHOR decomposition of cen8 into nine superHORs (bottom). cen8 is represented in the alphabet of 12 monomers. Different colors represent different superHORs. The length of the run-length encoding of the HOR (superHOR) sequence for cen8 is 893 (141). “NM” represents non-monomeric region.

Supplementary Note: Modifying HORDecomposer to incorporate canonical HORs

Previous studies manually inferred some human HORs that we refer to as *canonical* HORs (Shepelev et al., 2015, McNulty and Sullivan, 2018, Uralsky et al. 2019). To incorporate canonical HORs, we represent them in the monomer alphabet. Given a monocentromere *S* and a list of canonical HORs, we first add all canonical HORs to the list of HORs (with the corresponding transformation of the string *S*) and continue with the HORdecomposer algorithm in the same way as before. Tables cenXdecompositionCanonical, cen6decompositionCanonical, and cen8decompositionCanonical present modified results on HOR decomposition of cenX, cen6, and cen8, respectively.

HOR name	HOR length	HOR	Count	Weight	Run-length
a	12	ABCDEFGHIJKL	1471	17620	472
b	6	ABCDEF	28	1616	332
c	5	GHIJK	23	1574	234
d	17	bGHIJKL	6	1526	190
e	22	abGJKL	8	1522	152
f	17	HIJKLa	8	1515	119
g	3	CDE	7	1489	105
h	17	bccL	6	1487	93

cLa[115]ea[180]AgFcLa[33]d[3]a[2]da[61]bGgFcLa[144]ha[3]ha[15]ha[6]ha[4]ha[5]ha[41]ABgfABgfa[12]bfa[7]bfa[18]bfa[7]bfa[17]bfa[7]ABgfa[239]ABgLa[224]da[3]da[6]ea[10]e[4]a[10]eaea[128]ABg_LINE_FcLa[133]bca[24]bG

Table cenXdecompositionCanonical. The modified set of HORs (top) and decomposition of cenX into these HORs (bottom) that incorporates information about the canonical 12-monomer HOR in cenX. To generate this table, HORDecomposer was launched in a mode that selects the canonical 12-monomer HOR in cenX at the first step.

HOR name	HOR length	HOR	Count	Weight	Run-length
a	18	ABCDEFGHIJKLMNOPQR	649	11503	4807
b	15	ABFGHIJKLMNOPQR	281	4516	761
c	14	DEFGHIJKLMNOPQ	18	816	527
d	9	GHIJKLMNO	6	630	479
e	16	AcR	14	616	445
f	21	aABC	8	606	427
g	3	PQR	8	598	411

NOgARABHIEFdga[42]fcLMNOgea[3]ea[2]eae[3]a[4]fcLMNOgea[5]e[3]a[8]e[3]a[44]b[2]aba[15]b[2]a[2]b[2]ab[2]aba[10]fdga[6]ba[20]babababab[3]a[3]bab[2]eba[3]b[3]a[2]ba[3]ba[3]babafDEFGb[2]a[5]ba[12]ba[2]ba[11]fdgb[2]ab[2]a[2]b[3]abab[2]ab[3]a[2]b[8]ab[3]a[2]ABFdcRbab[3]ab[5]ab[3]ab[2]aba[2]b[4]a[3]b[2]ab[2]a[3]b[2]a[2]b[2]aba[2]ba[2]ba[2]ba[2]b[4]a[4]b[3]a[2]b[2]ababa[2]bab[2]ab[2]ababab[2]aba[2]baba[22]b[11]ab[4]a[2]b[3]ab[2]ab[3]a[2]b[10]ab[3]a[4]baba[2]b[3]a[3]b[2]aba[2]ba[2]b[2]a[2]ba[3]b[3]ab[2]ababa[2]bab[2]abab[3]abababababa[6]b[2]abababa[7]b[3]ab[4]a[3]ba[4]ba[4]baba[19]baba[4]ba[2]ba[5]ba[6]b[2]aba[5]baba[23]baba[4]baba[2]baba[8]baba[4]ba[2]ba[5]ba[6]baba[5]baba[24]baba[4]baba[3]baba[2]bababa[3]fdga[2]baba[2]b[2]a[3]ba[23]ba[6]baba[6]baba[3]ba[3]b[2]ababa[3]babfdgab[4]a[4]baba[6]ba[3]ba[3]bABFcRa[15]bababa[2]ba[5]baba[2]ba[8]b[4]a[4]b[2]a[5]b[4]aba[3]b[4]aba[2]babfD

Table cen6decompositionCanonical. The modified set of HORs (top) and decomposition of cen6 into these HORs (bottom) that incorporates information about the known 18-monomer HOR in cen6. To generate this table, HORDecomposer was launched in a mode that selects the canonical 18-monomer HOR in cen6 at the first step.

HOR name	HOR length	HOR	Count	Weight	Run-length
h1	15	ABCDEFGHIJKLMNO	27	378	11866
h2	11	GHIJKDMNOAB	347	3537	8333
h3	8	DEFGHIJK	278	2133	6267
h4	5	MNOAB	406	1811	4643
h5	5	GHIJK	198	979	3851
h6	15	DEFh2C	165	891	3147
h7	7	Lh4C	169	663	2715
h8	5	ENOAB	100	725	2315
h9	7	Dh4C	134	623	2017
h10	8	LEFh5	58	545	1827
h11	22	DMNOh1ABC	21	519	1679
h12	15	LEFh2C	24	468	1583
h13	11	h5Lh4	32	453	1502
h14	18	Dh4h2C	23	463	1428

h15	11	h5Dh8	28	465	1357
h16	6	LMFABC	11	465	1301
h17	14	DEFh2	21	465	1246
h18	15	Dh8Ch3	18	453	1195
h19	11	h5Lh8	20	448	1146
h20	11	Dh4h5	16	440	1114
h21	12	h2C	43	438	1084
h22	19	NOh1AB	6	419	1060
h23	6	Dh8	20	415	1040
h24	7	Lh8C	10	415	1020
h25	6	Lh4	19	414	1001
h26	11	h3DMF	6	412	984
h27	17	Dh4h2	8	409	969
h28	16	Ch6	6	398	964

ABh2[2]h15h19[2]h15h19[3]h15[7]h5LENONABh19[2]h5DKh23h15h19[2]h15h19[2]h15[2]
h19h5LENONABh5LENNABh19h5DKh23h15[8]h13h15[2]h2h15h19h15h5h11h25h5h3[2]h11[2]
h7h3h11h9h6h9h3[2]h7h9h3h6h3h17h2[5]h21h17h5h12h6[2]h9h11h9h3h17h5h10h7h3h24h
6h3h17h2h21h9h11h3h23h2h21h17h2[2]h21h3[10]N[3]EFh5h3[2]N[3]EFh5h3[9]h17h5h3[
6]h17h2[2]GJKDEFABCh9[2]h6h3h26OABCh3[2]h25h21h17h5h7h17h5h7h17h21h3h17h5h7h3
[6]h25h5h17h5h3[2]h25h5h3h10h25h5h11h3h6[3]h17h21h6h17h2h21h6[6]h3[2]DMh22h21
h6[2]h3h18[2]h3h18[2]h3h24h3DEh22Ch3h6h10h18h3[2]h24h3h10[2]h24h10h24h3h10[2]
h3h10[4]h3[2]h10[2]h3h10h3h10h3h23h28DMh22h5h7h6h3h24h3h12h6h3h18h10[2]h17h21
h7[2]h16h9h3h10h18h10[2]h3h10h18h3h10h18h10[2]h3h10[2]h3[2]h10[3]h3h10h7h3[2]
h12h3[2]h10[4]h3h7h3[3]h10h3h6h3h10[2]h3h6[2]h9h12h9LEFh2Gh9h3[3]h7h9h3[2]h6h
3[2]h6[2]h3[2]h7h3[2]h7h3h6[6]h3h11h6h3h11h3h11h12h6h12h3h6h3[3]h11DEFh28h6[3
]DEFh28h10h3h12h3[2]h7h3h6h3h6h3[3]h12LMFh21h6[2]h3h6h3h6[4]h3h11h3DMNIJKh9h6
[15]h3[2]h7h3[3]h7h3[3]h7h3[3]h7h3h11h3h12h6[4]h26h5h26h5h3h12h6h3h11h3h6[2]h
12h6h3[3]h11h10h3h12h3h18DMFh21h10h3h6h12h7[3]h10h3h10h3h6h12h7[3]h10h3h12h3h
23h28h12h9h3h18h23h28h3h12h10h18h3[2]h10h12h10h18h6h10h18h6h10h18h6h12h10h18h
12h10h18h6h12h10h18h6h12h3[2]h6h3h23h28h9h27h5DMBCh9[2]h7[2]h9[2]h25h2h21h9h1
4h9[4]h7h9h7DMFABCh14[2]h7h9[7]h16h9[6]h16h9[2]h7h16h9h7h25h21Dh4h13h21Lh8h5h
7Dh4h13h21h14h25h21h16h7h25h21h20h7h25h5h7Dh4h13h21h14h25h21h16h14[2]h7[2]h16
h9[2]h14h20h7h14Dh4h13h5h7h20h7h20h7h16h14h9[2]h16h9h14h25h13h21h9h14h9[3]h7h
9[2]h7h9[2]h7[3]h9[2]h7[5]h9[2]h7[2]h9[2]h7[7]h9h7[6]h9h7h9h7[13]h9h7[4]h9h7[
3]h9h7[14]h9h7[2]h9h7[14]h25h5h7h25h21h9h3[2]h7h9h14h7[2]h3[2]h7[3]h14h7h25h5
h7[3]h3[2]h7[2]h9h25h21h25h5h7[2]LMh22Ch7h9[2]h7LMFh21h7h9h7LMFh21h7h9h7[4]h9
h7h9[2]h7[4]h9h7[4]h9h7[5]h16[2]h9h7[2]LEh22Ch3[2]h6h9[2]h6h7h24h9h24h9h24h6h
3h6h7[3]h3h6h7[3]DEh22Ch24h6h10h3[2]h6h3[2]h6h3[2]h6h9h3[4]h6h11h25h5h6h23h21
h3h6h3[2]h6h9h3[3]h7h11h14h3[2]h6h9h3[2]h6h11h20h6h23h21Dh4h15Ch3[2]h6h9h3[2]
h6h23h21h3[2]h6h9h3h6h9h23h21h9h23h5h6h20h6h9h23h21h3[2]h6h3[2]h6h9h3h6h9h3[2
]h6h9h3[2]h10h6h9h3h6h9h3h6h9h3h6h9h26h5h17h5h6h23h5h6h23h2h21h3h6h20h6h27h5h
6[2]h9h6h9h3[3]h6h20h6h23h2h21h9h26h21h27h21h11h9h3h6h9h6h9h6h9h10h3[2]h6h14h
23h21h6[2]h20h6h14h27h5h6[2]h9h6h9h3[3]h6h20h6h23h2h21h9h26h21h27h21h9h6h9h3h
6h9h6h9h6h9h10h3[2]h6h14h23h21h6h11h9h6h9h6h9h3[3]h6h27h21h9h6h9h6h9h6h9h10h3
[2]h6h14h23h21h6h11h9h6h9h6h9h3[3]h6h27h21h9h6h9h6h9h6h9h3h6h9h6h9h6h9h3[3]h6
h20h6h14[4]h20h6h20h17h5h6h20h6h20h3[4]h6h20h7h12h3[2]h7h3DMNOACh12h25h21h9h3
[5]h17h5h3[5]h17h5h3[2]h17h5h3[3]h17h5h3[3]Lh8h19[2]h13h5h3Lh8h19[2]h5h7h10Lh
8h19[2]h13h2[23]h5DMNOIJKh27h2[7]h15h2[3]h15h13[2]h2[3]h13h2h13h2h13h2[3]h13[
14]h2h13[2]h2h13[3]GHGJGJKL

Table cen8decompositionCanonical. The modified set of HORs (top) and decomposition of cen8 into these HORs (bottom) that incorporates information about the canonical 15-monomer HOR in cen8. To generate this table, HORDecomposer was launched in a mode that selects the canonical 15-monomer HOR in cen8 at the first step. Each HOR is represented by a letter “h” and its ID.

Supplementary Note: Monomer inference for cen6 and cen8

MonomerGenerator generated monomer-sets of size 23 and 14 in cen6 and cen8, respectively (Tables S-MIC68-1 and S-MIC68-2). In order to compare the generated monomers with the reference monomers, we shifted the generated monomers by 77 nucleotides. In this section we compare only frequent monomers with the reference monomers set. Table S-MIC68-3 (S-MIC68-4) compares the nucleotide sequences generated by MonomerGenerator with reference monomers for cen6 (cen8).

18 (5) of the cen6 monomers are frequent (infrequent). After shifting and merging similar monomers, the transformed monomer-set for cen6 consists of 18 frequent monomers and 5 infrequent monomers. The frequent monomers correspond to the reference monomers that form the D6Z1 HOR (Dvorkina et al., 2020) and are abundant in the cen6 satellite array.

11 (3) of the cen8 monomers are frequent (infrequent). After shifting and merging similar monomers, the transformed monomer-set for cen8 consists of 12 frequent monomers and 6 infrequent monomers. The frequent monomers correspond to reference monomers that form the D8Z2 HOR (Dvorkina et al., 2020) and are abundant in the cen8 satellite array.

The reference monomer-set for cen8 contains 15 monomers, but the number of frequent monomers in the shifted monomer-set generated by MonomerGenerator is 12. Some reference monomers are very similar to each other: monomers D and L differ in a single nucleotide, monomers E and M (as well as F and N) differ in only 3 nucleotides. MonomerGenerator generated a single monomer for such similar pairs of monomers.

For cen6 and cen8, frequent monomers mostly coincide with the reference monomers. Some monomers have small gaps either at the start or at the end which can be explained by minor inconsistency of the shift selection between some reference monomers and frequent monomers. Some monomers have a small (at most six) number of mismatches/indels as compared to the reference monomers, which may be explained by inaccuracies in the reference monomers and centromere polymorphism across the population.

iteration	# resolved blocks	# unresolved blocks	# non-monomeric blocks	largest connected component	# removed monomers	radius	min distance to the previous monomers	length of new monomer
0	0	16315	0	956	1	5	100	169
1	0	16315	0	956	1	7	42	172
2	0	16315	0	955	1	6	41	171
3	0	16315	0	954	1	5	39	171
4	0	16315	0	954	1	5	29	169
5	0	16315	0	954	1	4	25	171
6	0	16315	0	954	1	6	29	170
7	6687	9628	0	954	0	6	31	170
8	6687	9628	0	953	0	5	16	169
9	6687	9628	0	952	0	4	27	171

10	6687	9628	0	952	0	6	27	169
11	10505	5810	0	953	0	5	16	171
12	10505	5810	0	951	0	7	23	168
13	10505	5810	0	940	0	5	21	171
14	10505	5810	0	669	0	10	18	169
15	10505	5810	0	669	0	5	28	169
16	10505	5810	0	656	0	5	22	167
17	15584	731	0	654	0	4	9	170
18	15584	731	0	45	0	3	8	169
19	16294	21	0	3	0	2	10	171
20	16294	21	0	2	0	0	12	171
21	16294	21	0	2	0	0	31	138
22	16301	14	0	2	0	2	10	163
23	16299	12	0	1	0	-	-	-

Table S-MIC68-1. Information about monomers generated MonomerGenerator for cen6.

iteration	# resolved blocks	# unresolved blocks	# non-monomeric blocks	largest connected component	# removed monomers	radius	min distance to the previous monomers	length of new monomer
0	0	12239	4	1514	1	7	34	171
1	1516	10709	18	1514	0	7	39	171
2	3030	9204	9	1510	0	8	52	167
3	4544	7695	4	1513	0	8	27	171
4	6044	6195	4	923	0	7	31	171
5	6969	5270	4	916	0	8	23	171
6	7895	4344	4	913	0	7	30	171
7	8808	3431	4	852	0	8	27	167
8	9661	2578	4	851	0	7	25	167
9	10513	1726	4	850	0	8	34	171
10	11366	873	4	844	0	7	27	170
11	12217	22	4	7	0	7	9	171
12	12230	9	4	2	0	4	62	113
13	12233	6	4	2	0	0	11	167
14	12235	4	4	1	0	-	-	-

Table S-MIC68-2. Information about monomers generated by MonomerGenerator for cen8.

generated monomer	reference monomer	divergence with the	number of occurrences

<table>
<tr><td></td><td></td><td>reference monomer</td><td></td></tr>
<tr><td colspan="4">frequent monomers</td></tr>
<tr><td>B-F</td><td>J_9_split_1000964_1004021_doubled/1474_1646/F</td><td>4.62</td><td>955</td></tr>
<tr><td colspan="4">-TCTGTCTAGTTTTTTATAGGAAGATGTTTCTTTTTTCTGCCATAGGCTCAATGCGCTATAAATATCCCCTTGGAAATCCTACAAAAAACAGTGTTTCAAAACTGCTCTGTGAAAAGGGAGGTTTCACTCTTTGAATTGAATGCACACATCACAAAGGAGTTTCTGAAAATTCT
-||||||||||||||-||||||||||||||||||||.|-|||.|||||||||||||||||||||||||||||||||||||||||||||-||||||||||||||||||||||||||||||||||||||||||||||||||||||||||||||||||||||||||||||.|||||-
TTCTGTCTAGTTTTT-ATAGGAAGATGTTTCTTTTTCC-GCCGTAGGCTCAATGCGCTATAAATATCCCCTTGGAAATCCTACAAAAA-CAGTGTTTCAAAACTGCTCTGTGAAAAGGGAGGTTTCACTCTTTGAATTGAATGCACACATCACAAAGGAGTTTCTGAGAATTC-</td></tr>
<tr><td>F-D</td><td>K_10_split_1000964_1004021_doubled/1647_1818/F</td><td>2.9</td><td>955</td></tr>
<tr><td colspan="4">TCTGTCTAGGTTTTAGGTGAAGTTATTTCCTTTTCTACTGTGGGCTTCAATGCGCTCTAAATATACACATGCAAATACTACAAAAAAGAGTGTTTCAAAACTGCTCTATCAAAAGAAAAGTTTTACTCTGTGGGTTGAACGCACACATCGCAAAGCAGATTCTGAGAATTAT
-|||||||||||||||||||||||||||||||||||||||||||||||||||||||||||||||||||||||||||||||||||||-|||||||||||||||||||||||||||||||||||||||||||||.|||||||||||||||||||||||||||||||||||.||-
-CTGTCTAGGTTTTAGGTGAAGTTATTTCCTTTTCTACTGTGGGCTTCAATGCGCTCTAAATATACACATGCAAATACTACAAAAA-GAGTGTTTCAAAACTGCTCTATCAAAAGAAAAGTTTTACTCTGTGAGTTGAACGCACACATCGCAAAGCAGATTCTGAGAAGTA-</td></tr>
<tr><td>D-E</td><td>L_11_split_1000964_1004021_doubled/1819_1988/F</td><td>2.33</td><td>957</td></tr>
<tr><td colspan="4">--TCTGTCTAGGGTTATAGGAAGAAATCCCGTTTCCAACGAAGGCCTCAAAGAGGTCCAAATATCCACTTGCAGTTTCTACAAAAAAGAGTGTTTCAACACTGCTCTATAAAGAGGAAAGTTCCACTCTGTGAGTTGAATGTACACATCACAAAGTAGTTTCTGAGATTGCT-
--||||||||||||||||||||||||||||||||||||||||||||||||||||||||||||||||||||||||||||||||||||-|||||||||||||||||||||||||||||||||||||||||||||||||||||||||||||||||||||||||||||||||||||-
CTTCTGTCTAGGGTTATAGGAAGAAATCCCGTTTCCAACGAAGGCCTCAAAGAGGTCCAAATATCCACTTGCAGTTTCTACAAAAA-GAGTGTTTCAACACTGCTCTATAAAGAGGAAAGTTCCACTCTGTGAGTTGAATGTACACATCACAAAGTAGTTTCTGAGATTGCTT</td></tr>
<tr><td>E-G</td><td>M_12_split_1000964_1004021_doubled/1989_2159/F</td><td>1.17</td><td>957</td></tr>
<tr><td colspan="4">TCTATCTATGTTTTCCATGAAGATGTTTCCTTTTCTATCATAGGCTTCAAAGTGGTCTAAATATCCACTTGGAAATCCTACAAGAACAGGGTTTCAAAACTTCTCTATCAAACGGAAGACTCCACTCTGTGAGATGAACGCACACATCACAATGAGGTTTCTGAAAATTCT
|||||||||||||||||||||||||||||||||||||||||||||||||||||||||||||||||||||||||||||||||||||||||||||||||||||||||||||||||||||||||||||||||||||||||||||||||||||||||||||||||||||||||--
TCTATCTATGTTTTCCATGAAGATGTTTCCTTTTCTATCATAGGCTTCAAAGTGGTCTAAATATCCACTTGGAAATCCTACAAGAACAGGGTTTCAAAACTTCTCTATCAAACGGAAGACTCCACTCTGTGAGATGAACGCACACATCACAATGAGGTTTCTGAAAATT--</td></tr>
<tr><td>G-H</td><td>N_13_split_1000964_1004021_doubled/2160_2330/F</td><td>1.75</td><td>957</td></tr>
<tr><td colspan="4">TCTGTCTAGCTTTTATGGAAAGATATTTCCTTTTCTACCATAGGCCTCAAAGCGCTCTTAGTATACACTTCCAAATTCTACAAAGAGAGTGTTACTAAACCGCTCTCTCAAAGGAAATGTTAAACTCTGTGAGTTGAACACAGACATCACAAAGCAGTTTCTGAGAACACT-
|||||||||||||||||||||||||||||||||||||||||||||||||||||||||||||||||||||||||||||||||||||||||.||||||||||||||||.||||||||||||||||||||||||||||||||||||||||||||||||||||||||||||||||-
TCTGTCTAGCTTTTATGGAAAGATATTTCCTTTTCTACCATAGGCCTCAAAGCGCTCTTAGTATACACTTCCAAATTCTACAAAGAGAGGGTTACTAAACCGCTCTATCAAAGGAAATGTTAAACTCTGTGAGTTGAACACAGACATCACAAAGCAGTTTCTGAGAACACTT</td></tr>
<tr><td>H-A</td><td>O_14_split_1000964_1004021_doubled/2331_2501/F</td><td>1.74</td><td>952</td></tr>
<tr><td colspan="4">TCTGTCTAGCTTTTATGGAAAGATATTTCCTTTTCTACCATAGGCCTCAAAGCGCTCTTAGTATACACTTCCAAATTCTACAAAGAGAGTGTTACTAAACCGCTCTCTCAAAGGAAATGTTAAACTCTGTGAGTTGAACACAGACATCACAAAGCAGTTTCTGAGAACACT-
|||||||||||||||||||||||||||||||||||||||||||||||||||||||||||||||||||||||||||||||||||||||||.||||||||||||||||.||||||||||||||||||||||||||||||||||||||||||||||||||||||||||||||||-
TCTGTCTAGCTTTTATGGAAAGATATTTCCTTTTCTACCATAGGCCTCAAAGCGCTCTTAGTATACACTTCCAAATTCTACAAAGAGAGGGTTACTAAACCGCTCTATCAAAGGAAATGTTAAACTCTGTGAGTTGAACACAGACATCACAAAGCAGTTTCTGAGAACACTT</td></tr>
<tr><td>A-I</td><td>P_15_split_1000964_1004021_doubled/2502_2670/F</td><td>1.18</td><td>952</td></tr>
<tr><td colspan="4">T-CTGTCTGGGTTTATAAGATGAAAACCCGTTTCCAACGAAGGCCTCAAGGAGGTCCAAATACAAACAAGCTGATTCTACAGAAAGAGTGTTTCCAAACTGCTCTATCAAGAGGAATGTTCCACTCGGTGAGTTGAATGCAGACATCACAAAGGAGTTTCTGAGATTGCT
|-|||||||||||||||||||||||||||||||||||||||||||||||||||||||||||||||||||||||||||||||||||||||||||||||||||||||.||||||||||||||||||||||||||||||||||||||||||||||||||||||||||||||||
TTCTGTCTGGGTTTATAAGATGAAAACCCGTTTCCAACGAAGGCCTCAAGGAGGTCCAAATACAAACAAGCTGATTCTACAGAAAGAGTGTTTCCAAACTGCTCTCTCAAGAGGAATGTTCCACTCGGTGAGTTGAATGCAGACATCACAAAGGAGTTTCTGAGATTGCT</td></tr>
<tr><td>I-J</td><td>Q_16_split_1000964_1004021_doubled/2671_2842/F</td><td>1.16</td><td>952</td></tr>
<tr><td colspan="4">TCTGTCTACCTTTTATGGAAAGATATTCCCTTTTCTACCATAGGCCTGAAAGCGCTCTCAATGTACCCTTGCAAATTCTACAAAAAAGAGTGTTTCCAAATTGCTCTATCAAGAGAAATCTTTATCTCGGTGAGTTGAAAGCACACATCACAAAGAAGACTCTGAGAATTCT
||||||||||||||||||||||||||||||||||||||||||||||||||||||||||||||||||||||||||||||||||||||-||||||||||||||||||||||||||||||||||||||||||||||||||||||||||||||||||||||||||||-
TCTGTCTACCTTTTATGGAAAGATATTCCCTTTTCTACCATAGGCCTGAAAGCGCTCTCAATGTACCCTTGCAAATTCTACAAAAA-GAGTGTTTCCAAATTGCTCTATCAAGAGAAATCTTTATCTCGGTGAGTTGAAAGCACACATCACAAAGAAGACTCTGAGAATTC-</td></tr>
<tr><td>J-K</td><td>R_17_split_1000964_1004021_doubled/2843_3011/F</td><td>3.53</td><td>952</td></tr>
<tr><td colspan="4">T-CTGTCTGGGTTTATAGGAAGAAATCCCGTTTCCAACGAAGGCCTCAAAGCGGTCCATATATCCACTTGCAGATTCTACAGAAACAATGTTTCCAAACTGCTCTATCAAGAGGAATGTTGCACTCGGTGAGTTGAATGCACACATCACAAAGTAGTTTCTGAGATTGCT
|-|||||||||||||||||||||||||||||||||||||||||||||||||.||||||.|||||||||||||||||||||||||||||||||||||||||||||..||||||||||.|||||||||||||||||||||||||||||||||||||||||||||||||||||
TTCTGTCTGGGTTTATAGGAAGAAATCCCGTTTCCAACGAAGGCCTCAAAGAGGTCCAAATATCCACTTGCAGATTCTACAGAAACAATGTTTCCAAACTGCTCGGTCAAGAGGAACGTTGCACTCGGTGAGTTGAATGCACACATCACAAAGTAGTTTCTGAGATTGCT</td></tr>
<tr><td>K-L</td><td>A_0_split_1000964_1004021_doubled/3012_3183/F</td><td>2.33</td><td>952</td></tr>
<tr><td colspan="4">TCTGTCTAGGTTTTATGGGAAGATATTTCCTTTTCTACCATACGCTTCAAGGCGTTCCAAATATCCGCTTGGAAATACTACAAAAAACAGTGTTTCAAAACTGCTCTATCAAAAGGAAGGATCCACACTGTGAGTTGAATTCACACATCACAAAGAAATCTCTGAGAATTCT
||||||||||||||||||||||||||||||||||||||||||||||||||||||||||||||||||||||||||||||||||||||-|||.||||||||||||||||||||||||||||||||||||||||||||||||||||||||||||||||||.|||||||||||||-
TCTGTCTAGGTTTTATGGGAAGATATTTCCTTTTCTACCATACGCTTCAAGGCGTTCCAAATATCCGCTTGGAAATACTACAAAAA-CAGGGTTTCAAAACTGCTCTATCAAAAGGAAGGATCCACACTGTGAGTTGAATTCACACATCACAAAGAAGTCTCTGAGAATTC-</td></tr>
<tr><td>L-M</td><td>B_1_split_1000964_1004021_doubled/119_286/F</td><td>2.35</td><td>937</td></tr>
</table>

T-CTGTCTGGATTTATACGAAGAAATCCCGTTTCCAACGAAGACCCAAAGGAGTTCCAAATATCCACTTGCAGATCCTTCAGAAAGAGGGTTTCAAAACTGCTCTATCAAGAGAAATGTTCAACTCTGTGAGTTGAATGCAGACATCACAAAGTCGTTTCTGAGATTGG- |-||||||.||||||||||||||||||||||||||||||||||||||||||||||||||||||||||||||||||||||||||||||||||||||||||||||||||||||||||||||||||||||.||||||||||||||||||||||||||||||||||||-<br>TTCTGTCTAGATTTATACGAAGAAATCCCGTTTCCAACGAAGACCCAAAGGAGTTCCAAATATCCACTTGCAGATCCTTCAGAAAGAGGGTTTCAAAACTGCTCTATCAAGAGAAATGTTCAACTCTGTGACTTGAATGCAGACATCACAAAGTCGTTTCTGAGATTGGT			
M-N	C_2_split_1000964_1004021_doub led/287_454/F	1.79	669
T-CCGTCTAGGTTTTATGGGAAGATATTTCCTTTTCTACCATAGGCCTCAAGGCGCTCTAATATCCGCTTGGAAATACTACAACCACAGCGTTTCAAACTGCTCTATCCAAAGGAAGGTTCCACTCTGTGACTTGAATGCACACAACCAAAGAAGTTTCGGAGAATTCT |-||||||||||||||||||||||||||||||||||||||||||||||||||||||||||||||||||||||||||||||||||.|||||||||||||||||||||||||||||||||||||||||||||||||||||||||||||||||||||||||||||||||||||||-<br>TTCCGTCTAGGTTTTATGGGAAGATATTTCCTTTTCTACCATAGGCCTCAAGGCGCTCTAATATCCGCTTGGAAATACTACAACCACGGCGTTTCAAACTGCTCTATCCAAAGGAAGGTTCCACTCTGTGACTTGAATGCACACAACCAAAGAAGTTTCGGAGAATTC-			
N-P	D_3_split_1000964_1004021_doub led/455_623/F	1.77	669
T-CTGTCTAGAGTAACATGAAGAAATCCCGTTTCCAACGAAGGCCTCAAGGCGGTCCAATTATCCACTTGCAGATTCTACAGAAAGAGTGTTTCAAAACTGCTCTATCAAGAGAAATGTTCCACCGTGTGTGTGGAATGCAGCCATCACACAGTAGTTTCTGAGATTGCT |-||.|||||||||||||||||||||||||||||||||||||||||||||||||||||||||||||||||||||||||||||||||||||||||||||||||||||||||||||||||||||||||||||||||||||||||||||||||||||||||||||||||||-<br>TTCTATCTAGAGTAACATGAAGAAATCCCGTTTCCAACGAAGGCCTCAAGGCGGTCCAATTATCCACTTGCAGATTCTACAGAAAGAGTGTTTCAAAACTGCTCTATCAAGAGAAATGTTCCACCGTGTGTGTGGAATGCAGCCATCACACAGTAGTTTCTGAGATTGC-			
P-O	E_4_split_1000964_1004021_doub led/624_792/F	2.37	669
T-CTGTCTAGATTCATACGAAGAAATCCCGTTTCCAACGAAGGCCTCAAAGAAGTCCAAATATCCCATTGCAAATTCTACAAAAGGAGTGTTTCCCAACTGCTCTATCAAGAGGAATGTTGCACTCTGTGACTTGAATGCAAACATCACATAGCAGTGTTTGAGAATTCT |-||||||||||||||||||||||||||||||||||||||||||||||||||||||||||||||||||||||.|||||||||||||||||||||||||||||||||.|||||||||||||||||||||||||||||||||||||||||||||||||||||||||||||||||||||||||-<br>TTCTGTCTAGATTCATACGAAGAAATCCCGTTTCCAACGAAGGCCTCAAAGAAGTCCAAATATCCCATTGCAGATTCTACAAAAGGAGTGTTTCCCAACTGCTCTACCAAGAGGAATGTTGCACTCTGTGACTTGAATGCAAACATCACATAGCAGTGTTTGAGAATTC-			
O-R	F_5_split_1000964_1004021_doub led/793_964/F	1.16	954
TCTGTCTTGGTTTTATGGGGAGATATTTCCATTTCTAGCATAGGCTTCAAGGCGCTCTAAATATCCGCTTGGAAATACTACAAAAAACAGTGTTTCAAAACTGCTGTATCCAAAGGAAGGTGCCACTCGCTGAGTTGAATGCACACATCACAAGGAAGTTTCTGAGAATTCT ||||||||||||||||||||||||||||||||||||||||||||||||||||||||||||||||||||||||||||||||||-||||||||||||||||||||||||||||||||||||||||||||||||||||||||||||||||||||||||||||||||||||||||||||||||-<br>TCTGTCTTGGTTTTATGGGGAGATATTTCCATTTCTAGCATAGGCTTCAAGGCGCTCTAAATATCCGCTTGGAAATACTACAAAAA-CAGTGTTTCAAAACTGCTGTATCCAAAGGAAGGTGCCACTCGCTGAGTTGAATGCACACATCACAAGGAAGTTTCTGAGAATTC-			
R-S	G_6_split_1000964_1004021_doub led/965_1133/F	0.59	954
T-CTCTGTAGGTTTAGATGAAGAAATCCCGTTTCCAACGAAGGCCTCTAGGAGGTCCAATTATCCACTTGCAGATTCTACAGAAAGAGTGTTTCAAAACTGCTCTATCAAGAGAAATGGTCCACCGTGTGTGTGGAATGCAGCCATCACACATTAGTTTCTGAGATTGCT |-|||||||||||||||||||||||||||||||||||||||||||||||||||||||||||||||||||||||||||||||||||||||||||||||||||||||||||||||||||||||||||||||||||||||||||||||||||||||||||||||||||||||||||||| TTCTCTGTAGGTTTAGATGAAGAAATCCCGTTTCCAACGAAGGCCTCTAGGAGGTCCAATTATCCACTTGCAGATTCTACAGAAAGAGTGTTTCAAAACTGCTCTATCAAGAGAAATGGTCCACCGTGTGTGTGGAATGCAGCCATCACACATTAGTTTCTGAGATTGCT			
S-C	H_7_split_1000964_1004021_doub led/1134_1304/F	1.75	954
TCTATCTACTTTTTTACATGAAGATGTTTCCTTTTCTAGCAGAGACTTCAAAGTGCTCTAAATATCCACTTGGGAATTCTACAAAAACGGTGTCTCAAAACTGCTCTATCAAAGGGAATGTTCCATTCTGTGAGTCGAATGCACACATCCGAAGAAGTTACTGAGAATTCT |||||||||||||-||||||||||||||||||||||||||||||||||||||||||||||||||||||||||||||||||||||||||||||||||||||||||||||||||||||||||||||||||||.||||||||||||||||||||||||||||||||||||||-<br>TCTATCTACTTTT-ACATGAAGATGTTTCCTTTTCTAGCAGAGACTTCAAAGTGCTCTAAATATCCACTTGGGAATTCTACAAAAACGGTGTCTCAAAACTGCTCTATCAAAGGGAATGTTCCATTCTGTGCGTCGAATGCACACATCCGAAGAAGTTACTGAGAATTC-			
C-B	I_8_split_1000964_1004021_doub led/1305_1473/F	2.35	954
T-CAATCTAGAGTTACATGAAGAAATCCCGTTTCCAAAGAAGGCCTCAAATAGGTCCAAATATCCACTTGCAGCTACTACAAGAAGGGTGTTTCAGAAACGCTCTATCAAAAGAAACGTTAAACTCTGTGAGTTGAACACACACGTCACTAAGCACTTTCTGAGAACGAT |-||||||||||||||||||||||||||.||||||||||||||||.|||||||||||||||||||||||||||||||||||||||||||||||||||||||||||||||||||||||||||||||||||||||||||||||||.|||||||||||||||||||||||||||||||||| TTCAATCTAGAGTTACATGAAGAAATCCCATTTCCAAAGAAGGCCTCCAATAGGTCCAAATATCCACTTGCAGCTACTACAAGAAGGGTGTTTCAGAAACGCTCTATCAAAAGAAACGTTAAACTCTGTGAGTTGAACGCACACGTCACTAAGCACTTTCTGAGAACGAT			

Table S-MIC68-3. Comparing the shifted monomer-set generated by MonomerGenerator with the reference monomers from cen6.

generated monomer	reference monomer	divergence with the reference monomer	number of occurrences
**frequent monomers**			
E-F	A	0	925
TCTGTCTAGTTTTTGTCGGAAGATATTTCCTTTTTCAGCATAGGCCCCAAGGAGCTCAAAATGTCCACTGCCAGATAGTACGAGAAGATTGTTTCAAACCTGCTCTGTGAAAGGGAATGTTCAACTCTGTGACTTGAATGTAAACATCCCTAAGATGTTTCTTAGAATGCT ||||||||||||||||||||||||||||||||||||||||||||||||||||||||||||||||||||||||||||||||||||||||||||||||||||||||||||||||||||||||||||||||||||||||||||||||||||||||||||||||||||||||||||| TCTGTCTAGTTTTTGTCGGAAGATATTTCCTTTTTCAGCATAGGCCCCAAGGAGCTCAAAATGTCCACTGCCAGATAGTACGAGAAGATTGTTTCAAACCTGCTCTGTGAAAGGGAATGTTCAACTCTGTGACTTGAATGTAAACATCCCTAAGATGTTTCTTAGAATGCT			
F-G	B	0	925
TCTGTCTAGATTTCTTTGAAGACATTACCGTTTCCAACGAATCCTCAAAGCTAGCCAAATATCCACCTGCAGATTCTACAAAAAGAGTGTTTCAAAAGTGCTCTGTCCAAACCAAGGTTCAATTCTGACAGTTGAGTGCACACATCACAAACGTGATTCTGCGAATGCT |||||||||||||||||||||||||||||||||||||||||||||||||||||||||||||||||||||||||||||||||||||||||||||||||||||||||||||||||||||||||||||||||||||||||||||||||||||||||||||||||||||||||||| TCTGTCTAGATTTCTTTGAAGACATTACCGTTTCCAACGAATCCTCAAAGCTAGCCAAATATCCACCTGCAGATTCTACAAAAAGAGTGTTTCAAAAGTGCTCTGTCCAAACCAAGGTTCAATTCTGACAGTTGAGTGCACACATCACAAACGTGATTCTGCGAATGCT			

<table>
<tr><td>G-A</td><td>C</td><td>1.17</td><td>660</td></tr>
<tr><td colspan="4">TCTATCTCGCATTCATGGGAAGATATTTCCTTTTTCCAGATAGGCTACAAAGCCCTCCAAATGTCCACTTCCAGATACTACAAAAAGAGTGTTTCCAACCTGCTCTATGAAACGGAAGGTTCAACTCTGTGACTTGATTGCAAACATCACGACGCTGTTTCTGAGAATGCT
||||||||||||||||||||||||||||||||||||||||||||||||||||||||||||||||||||||||||||||||||||||||||||||||||||||||||||||||||||||||||||||||||||||||||||||||||||||||||.|.||||||||||||||||
TCTATCTCGCATTCATGGGAAGATATTTCCTTTTTCCAGATAGGCTACAAAGCCCTCCAAATGTCCACTTCCAGATACTACAAAAAGAGTGTTTCCAACCTGCTCTATGAAACGGAAGGTTCAACTCTGTGACTTGATTGCAAACATCACGAAGGTGTTTCTGAGAATGCT</td></tr>
<tr><td>A-B</td><td>D|L</td><td>2.34; 2.92</td><td>1513</td></tr>
<tr><td colspan="4">-GCTG-T--CTACTTTTTATATATAATCCCGTTTCCAACGAAATCCTCAAATCTATCCAAATATCCACTTGCAGATTCCAAAAGAAGAGTGTCTCAAAACTGCTCTATCAATAGAAATGTTCAGCACAGTTAGTTGAGTAGATACAGCATAAACATGTTTCTGAGATTACT
-||||-|--||||||||||||||||||||||||||||||||||||||||||||||||||||||||||||||||||||||||||||||||||||||||||||||||||||||||||||||||||||||||||||||||||||||||||||||||||||||||||
TGCTGCTGTCTACTTTTTATATATAATCCCGTTTCCAACGAAATCCTCAAATCTATCCAAATATCCACTTGCAGATTCCAAAAGAAGAGTGTCTCAAAACTGCTCTATCAATAGAAATGTTCAGCACAGTTAGTTGAGTAGATACAGCATAAACATGTTTCTGAGATTACT
|
-GCTG-T--CTACTTTTGATATATAATCCCGTTTCCAACGAAATCCTCAAATCTATCCAAATATCCACTTGCAGATTCCAAAAGAAGAGTGTCTCAAAACTGCTCTATCAATAGAAATGTTCAGCACAGTTAGTTGAGTAGATACAGCATAAACATGTTTCTGAGATTACT
-||||-|--||||||||.|||||||||||||||||||||||||||||||||||||||||||||||||||||||||||||||||||||||||||||||||||||||||||||||||||||||||||||||||||||||||||||||||||||||||||||||
TGCTGCTGTCTACTTTTTATATATAATCCCGTTTCCAACGAAATCCTCAAATCTATCCAAATATCCACTTGCAGATTCCAAAAGAAGAGTGTCTCAAAACTGCTCTATCAATAGAAATGTTCAGCACAGTTAGTTGAGTAGATACAGCATAAACATGTTTCTGAGATTACT</td></tr>
<tr><td>B-C</td><td>E|M</td><td>3.5; 2.92</td><td>1511</td></tr>
<tr><td colspan="4">TCTGTCTAGTTTCTGTAGGTAGATATTTCCTTTTTAAGCATAGGCCTGAAAGCGCTCCAAATGCCCGCTTCCAGACACTATAAAAAGAGGGTTTCAAACCTACTCTATGAAAGGGAATGTTCAACTCTGAGAGCTGGATGCAAACATCACAAAGAAGTTTCTGAGAATGCT
||||||||||||||.||||||||||||||||||||.|||||||||||||||||||||||||||||||||||||||||||||||||||||||||||||||||||||||||||||||||||||||||||||||||||||||||||||||||||||||||||||----
TCTGTCTAGTTTCTATAGGTAGATATTTCCTTTTTCAGCATAGGCCTGAAAGCGCTCCAAATGCCCGCTTCCAGACACTATAAAAAGAGGGTTTCAAACCTACTCTATGAAAGGGAATGTTCAACTCTGAGAGCTGGATGCAAACATCACAAAGAAGTTTCTGAGAA----
|
TCTGTCTAGTTTCTATAGGTAGATATTTCCTTTTTCAGCATAGGCCTGAAAGCGCTCCAAATGCCCGCTTCCAGACACTATAAAAAGAGGGATTCAAACCTACTCTATGAAAGGGAATGTTCAACTCTGAGAGCTGGATGCAAACATCACAAAGAAGTTTCTGAGAATGCT
|||||||||||||||||||||||||||||||||||||||||||||||||||||||||||||||||||||||||||||||||||||||||||.|||||||||||||||||||||||||||||||||||||||||||||||||||||||||||||||||||||||||||----
TCTGTCTAGTTTCTATAGGTAGATATTTCCTTTTTCAGCATAGGCCTGAAAGCGCTCCAAATGCCCGCTTCCAGACACTATAAAAAGAGGGTTTCAAACCTACTCTATGAAAGGGAATGTTCAACTCTGAGAGCTGGATGCAAACATCACAAAGAAGTTTCTGAGAA----</td></tr>
<tr><td>C-D</td><td>F|N</td><td>1.75; 0</td><td>1500</td></tr>
<tr><td colspan="4">TCTGTCTAGATTTTCTTTGAAGACATTCCCGTTTCCAACGAAATCCTCACAGCTATCCAAATATCCTCTTGCAGATTCTACAAAAAGTGTGGTTCAAAACTGCTGTATCAAAAGAATGGATCAACACTGTTAGTTGAGTACCCACATCACAAACGTGATTCTCAGAATGCT
||||||||.|||||||.|||||||||||||||||||||||||||||||||||||||||||||||||.||||||||||||||||||||||||||||||||||||||||||||||||||||||||||||||||||||||||||||||||||||||||||||||||||||||||
TCTGTCTAAATTTTCTATGAAGACATTCCCGTTTCCAACGAAATCCTCACAGCTATCCAAATATCCACTTGCAGATTCTACAAAAAGTGTGGTTCAAAACTGCTGTATCAAAAGAATGGATCAACACTGTTAGTTGAGTACCCACATCACAAACGTGATTCTCAGAATGCT
|
TCTGTCTAAATTTTCTATGAAGACATTCCCGTTTCCAACGAAATCCTCACAGCTATCCAAATATCCACTTGCAGATTCTACAAAAAGTGTGGTTCAAAACTGCTGTATCAAAAGAATGGATCAACACTGTTAGTTGAGTACCCACATCACAAACGTGATTCTCAGAATGCT
|||||||||||||||||||||||||||||||||||||||||||||||||||||||||||||||||||||||||||||||||||||||||||||||||||||||||||||||||||||||||||||||||||||||||||||||||||||||||||||||||||||||||||||||
TCTGTCTAAATTTTCTATGAAGACATTCCCGTTTCCAACGAAATCCTCACAGCTATCCAAATATCCACTTGCAGATTCTACAAAAAGTGTGGTTCAAAACTGCTGTATCAAAAGAATGGATCAACACTGTTAGTTGAGTACCCACATCACAAACGTGATTCTCAGAATGCT</td></tr>
<tr><td>L(D+G)-H</td><td>G</td><td>0</td><td>851</td></tr>
<tr><td colspan="4">TCTATGTCGTTTTTATGGGAAGATATTTCCTTTTTCACCATAGGCCTGAAAGCGCTCCAAATGTCCACTTCCAGATACTACAATAAGAGTGTTTCCAACCTGCTCTATGAAACGGAAGGTTCAACTCTGTGACTTGATTGCAAACATCACGAAGGTGTTTCTGAGAATGCT
|||||||||||||||||||||||||||||||||||||||||||||||||||||||||||||||||||||||||||||||||||||||||||||||||||||||||||||||||||||||||||||||||||||||||||||||||||||||||||||||||||||||||||||||
TCTATGTCGTTTTTATGGGAAGATATTTCCTTTTTCACCATAGGCCTGAAAGCGCTCCAAATGTCCACTTCCAGATACTACAATAAGAGTGTTTCCAACCTGCTCTATGAAACGGAAGGTTCAACTCTGTGACTTGATTGCAAACATCACGAAGGTGTTTCTGAGAATGCT</td></tr>
<tr><td>H-I</td><td>H</td><td>3.52</td><td>850</td></tr>
<tr><td colspan="4">-GCTG-TGT--ACGTTTTATATTGCATCCCGTTTCCAACGAAATCCTCAAAGCGATCCAAATATCCACTTGCAGATTCCAAAAAAAGAGTGTTTCAAACTGCTCTGTCAGTACAAACCTTCAACACTGTTAGTTGATTAGATGCATCATAAACAAGTTCCTGAGATAGCT
-||||-|||--|||||||||||||||||||||||||||||||||||||||||||||||||||||||||||||||||||||||||||||||||||||||||||||||||..||||||||||||||||||||||||||||||||||||||||||||||||||||
TGCTGCTGTGTACGTTTTATATTGCATCCCGTTTCCAACGAAATCCTCAAAGCGATCCAAATATCCACTTGCAGATTCCAAAAAAAGAGTGTTTCAAACTGCTCTGTCAGTACAAAGGTTCAACACTGTTAGTTGATTAGATGCATCATAAACAAGTTCCTGAGATAGCT</td></tr>
<tr><td>I-J</td><td>I</td><td>4.68</td><td>852</td></tr>
<tr><td colspan="4">TCTGTCTTGTTTTCATTGGAAGATATTTCCTTTTTCACCATAGTTCAGAAAGCGCTCCAAATGTCCACTTCCAGATACTCCAAAAAGAGTGTTTCAAACCTGCTCTATGAATCCCAATGTTCCACTCTGTGACTTGAATGGAAATATGGCAAAGTATTTTCTGAGTATGCT
|||||||||||||||||||||||||||||||||||||||||||||||||||||||||||||||||||||||||||||||||||||||||||||||.||||||||||||||||...||||||||||||||||||||||||||||||||||||||||||||||||||||----
TCTGTCTTGTTTTCATTGGAAGATATTTCCTTTTTCACCATAGTTCAGAAAGCGCTCCAAATGTCCACTTCCAGATACTCCAAAAAGAGTGTTTCCAACCTGCTCTATGAATGGGAATGTTCCACTCTGTGACTTGAATGGAAATATGGCAAAGTATTTTCTGAGTA----</td></tr>
<tr><td>J-K</td><td>J</td><td>2.34</td><td>853</td></tr>
<tr><td colspan="4">-GCTG-T--CTAATTTTTACATGTAAGCCCGTTTCCAACGAAATCCTCAAAGCTATCCAAATATCCGCATGCAGAATCTTCAAAAAGAGTGTTCCAGAAGTACTGCATGAAACGAAAGGTTCAAGTCCGTTTGTTGAGGACACACATCACAAATAAGTTTCTCAGAATGCT
-||||-|--||||||||||||||||||||||||||||||||||||||||||||||||||||||||||||||||||||||||||||||||||||||||||||||||||||||||||||||||||||||||||||||||||||||||||||||||||||||||||
TGCTGCTGTCTAATTTTTACATGTAAGCCCGTTTCCAACGAAATCCTCAAAGCTATCCAAATATCCGCATGCAGAATCTTCAAAAAGAGTGTTCCAGAAGTACTGCATGAAACGAAAGGTTCAAGTCCGTTTGTTGAGGACACACATCACAAATAAGTTTCTCAGAATGCT</td></tr>
<tr><td>K-A</td><td>K</td><td>2.34</td><td>853</td></tr>
<tr><td colspan="4">TCTATCTCGCATTCATGGGAAGATATTTCCTTTTTCCAGATAGGCTACAAAGCCCTCCAAATGTCCACTTCCAGATACTACAAATAGAGTGCTGCACAACTGCTCTATGTGAGGGGAAGTTCAATTCTGTGACTTGAATGCAGACACCACAAAGAAGTTTCTGAGAATGCT
|||||||||||||||||||||||||||||||||||||||||||||||||||||||||||||||||||||||||||||||||||||||||||||||||||||||||||||||||||||||||||||||||||||||||||||||||||||||||||||||||||||||----
TCTATCTCGCATTCATGGGAAGATATTTCCTTTTTCCAGATAGGCTACAAAGCCCTCCAAATGTCCACTTCCAGATACTACAAATAGAGTGCTGCACAACTGCTCTATGTGAGGGGAAGTTCAATTCTGTGACTTGAATGCAGACACCACAAAGAAGTTTCTGAGAA----</td></tr>
<tr><td>D-E</td><td>O</td><td>5.08</td><td>911</td></tr>
<tr><td colspan="4">TCTGGCTAGATTTTATTTGAAGATATTCCCGTTTCCAACGAAATCTTCAAAGCTTAAGCTTTCCAAATATCGACTTCCAGATTCTATAAAAAGAATGTTTCAGAACAGTTCTGTCAAAAGAAAGGTTCAACTCTGTTAGTGGAGAACACACATCACAATCAAGGTTCTGAGAATGCT
|||||||||||||.|||||||||||||||||||||||||||||||.|||||||||------||||||||||.|||||||||||||||||||||||||||||||||||||||||||||||||||||||||||||||||||||||||||||||||||||||||||||||||
TCTGGCTAGATTTGATTTGAAGATATTCCCGTTTCCAACGAAATCCTCAAAGCTT------TCCAAATATCCACTTCCAGATTCTATAAAAAGAATGTTTCAGAACAGTTCTGTCAAAAGAAAGGTTCAACTCTGTTAGTGGAGAACACACATCACAATCAAGGTTCTGAGAATGCT</td></tr>
</table>

Table S-MIC68-4. Comparing the shifted monomer-set generated by MonomerGenerator with the reference monomers from cen8.

Supplementary Note: Generating submonomers for cenX

Aligning *M*-blocks reveals highly mutated positions in monomers. For each monomer *M*, we aligned all its *M*-blocks to the consensus of *M*. This alignment reveals mutated positions in each monomer and enables downstream evolutionary analysis. Table

NumberMutationsInMonomers shows the number of mutations at each of 170 positions in the A-blocks for the monomer A in cenX and reveals some highly-variable positions within this monomer.

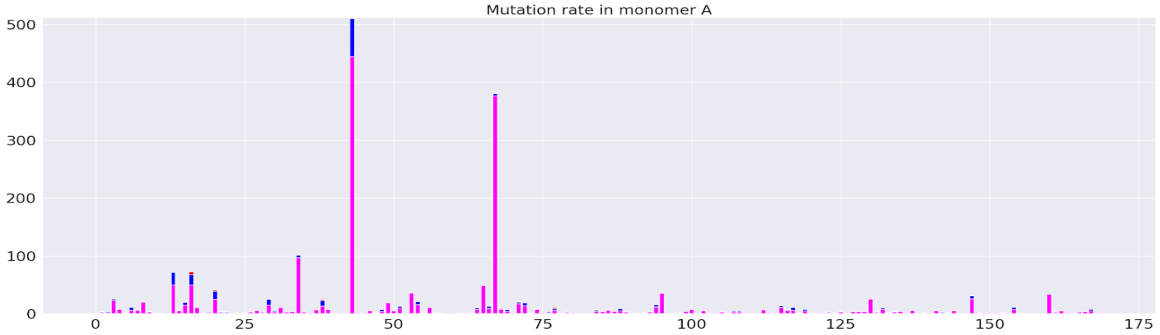

Table NumberMutationsInMonomers. The number of mutations at each of 170 positions of the A-blocks of the monomer A in cenX. The number of mutations is inferred from alignments of all 1505 A-blocks in cenX (591 of these A-blocks have length 170 nucleotides). Magenta, blue, and (hardly visible) red sub-bars represent the number of mutations into second, third, and fourth most frequent nucleotides at each position.

Identifying subfamilies of monomers. Given a position in a multiple alignment, we classify the most frequent nucleotide at this position as *dominant*. A nucleotide at this position is classified as rare if its count falls below a threshold *minCount* (the default value 5).

Given a monomer-set *Monomers* and a string *Centromere*, our approach to generating submonomers starts from launching StringDecomposer (Dvorkina et al., 2020) to generate the block-set *Blocks*(*StringDecomposer*,*Monomers*) and performing the following steps for each monomer *M* from *Monomers*:

1. Align all *M*-blocks to the consensus of monomer *M.*
2. Substitute each rare nucleotide in each position of the multiple alignment by the dominant nucleotide at this position.
3. Merge the resulting identical *M*-blocks into a single cluster and classify a cluster as *large* if its size exceeds *minClusterSize* (default value *minClusterSize*=20).
4. Define submonomers as consensuses of large clusters and define the *multiplicity* of a submonomer as the number of *M*-blocks in the cluster.

Table Submonomers presents the summary information about submonomers in cenX.

monomer	#clusters (#submonomer-instances in all clusters)	#large clusters with size > 20 (#submonomer-instances in all large clusters)	minimum distance between two large clusters	median distance between two large clusters
M(H/J)	1(8)	1(8)	-	-
E	122(1503)	10(1124)	1	3
H	138(1506)	8(1007)	1	2
B	148(1504)	11(1059)	1	2
C	124(1503)	13(1157)	1	3
G	94(1510)	9(1171)	1	3
J	88(1499)	10(1101)	1	2

A	165(1504)	14(1017)	1	3
D	59(1502)	7(1271)	1	2
K	91(1506)	7(1095)	1	2
F	84(1502)	8(1222)	1	3
I	75(1506)	8(1235)	1	2
L	89(1502)	9(1179)	1	3
N(J/K)	1(8)	1(8)	-	-

Table Submonomers. Summary of submonomer clusters in cenX. There are twelve canonical monomers and two hybrid monomers for cenX.

Supplementary Note: HOR hierarchy

Alexandrov et al. 2001, Shepelev et al. 2015, McNulty and Sullivan 2018, Uralsky et al., 2019, and Miga 2020 used the following levels of HOR hierarchy.

- *Suprachromosomal Family HORs* (SFs). An SF is a group of related HORs that share the same classes of monomers as defined by the branches in a phylogenetic tree of monomers (with divergence 10-20%). For example, SF3 includes live HORs of chromosomes 11, 17 and X, and a dead HOR from chromosome 1 (Alexandrov et al. 2001), while SF1 and SF2 cover the rest of the live HORs in all chromosomes except the Y (Alexandrov et al. 2001).
- *sub-SFs.* sub-SFs further sub-partition each SF and feature ~7-10% divergence within a sub-SF (Uralsky et al. 2019).
- *sister HORs*. Sister HORs are distinct (nucleotide) sequence variants (SqVs) of the same HOR which form smaller arrays adjacent to the live HOR. Sister HORs are formed by monomers which only slightly differ from monomers in the main HOR (divergence ~3-7%), and may have the same or slightly different order of monomers (e.g. D17Z1, D17Z1-B and D17Z1-C in Shepelev et al. 2009).
- *HOR haplotypes*. Durfy and Willard 1989 identified different haplotypes of the same HOR (SqVs) which occupy different regions in the live HOR domain. Divergence between HORs from different haplotypes varies in the range ~1-3% and divergence within a single haplotype varies in the range 0.5-1% (Logsdon et al. 2020).

Supplementary Note: Parameters of CentromereArchitect

CentromereArchitect parameter selection is an important and difficult task since it is the first study of completely assembled human centromeres and since previous studies provided limited information about parameters for monomer and HOR inference. Below we describe the rationale behind selection of CentromereArchitect parameters and current limitations for their selection.

MonomerGenerator parameters

- *Frequent, infrequent, and rare monomers.* The definition of a frequent and infrequent monomer is parametrized with the parameter *FreqCeiling*. Its default is set to 40, since all (canonical) human HORs inferred in previous studies are shorter than 40 monomers (the longest canonical HORs of cen4 is formed by 19 monomers). The rationale of this definition is that a frequent monomer has to appear in many (or most) HORs for a single chromosome. The definition of a rare monomer is parametrized with *rareMonomerCount* which, by default, is set to 3. Even though this choice is not

statistically justified, it does not affect MonomerGenerator performance and is only needed for the illustration purposes and descriptive analysis of its results.

- *Resolved and unresolved blocks.* The maximum divergence for a resolved block is *maxResolvedDivergence* which, by default, is set to 5%. Since most blocks have a rather conserved length ~ 171bp, the default roughly corresponds to 10 bp divergence. We selected *maxResolvedDivergence* as a conservative upper bound based on the observation that the average divergence of blocks generated by launching StringDecomposer on cenX with reference monomers is 2%. Similarly, we tuned the threshold *maxDivergence* (default is set to 40%) in the definition of a non-monomeric block. Specifically, we computed divergence 45.5% of blocks generated for decomposition of the single LINE element in cenX using the consensus alpha satellite.

- *Monomer-set resolves a centromere.* The threshold *FractionResolvedBlocks* is introduced to account for potential non-monomeric blocks in the definition of monomer-set that resolves a centromere. For example, in the decomposition generated by MonomerGenerator the LINE element in cenX corresponds to 38 non-monomeric blocks out of 18091 total blocks (0.2 %). The default *FractionResolvedBlocks* is selected as a reasonable lower bound. *MaxLengthDivergence* is set to 0.03**Length* since the length of blocks is rather conserved (mean 171 bp, standard deviation 4.7 bp). Note that this parameter is only required for formulating the Monomer Inference Problem, but is not explicitly used by the heuristic MonomerGenerator algorithm.

- *Hybrid monomers.* We select a rather low threshold *MaxHybridDivergence* (default value 1%), in order to be very conservative in reporting hybrid monomers. More experiments with various values of that parameters are required to comprehensively study the hybrid monomers phenomenon.

HORDecomposer parameters

- *Heavy substrings.* The defaults for thresholds *MinCount*, *MaxLength*, and *MinWeight* were selected through a thorough benchmark of HORDecomposer for various values of these parameters. All three parameters present a trade-off between reporting very rare or very long HORs and being too stringent, i.e., reporting only the most frequent HORs. For example, a radical selection *MinCount* = 1, *MaxLength* = 1000, *MinWeight* = 1 will lead to covering essentially the whole monocentromere with HORs, even though many of them may appear to be biologically meaningless. Alternatively, a conservative strategy *MinCount* = 50, *MaxLength* = 20, *MinWeight* = 100 will likely produce only primary HORs missing most secondary HORs.

- *Frequent, infrequent, and rare HORs.* Tuning parameters *HORFreqCeiling*, and *rareHORCount* is similar to selection of defaults for analogous monomeric parameters — *FreqCeiling,* and *rareMonomerCount.*

- *superHORs.* Since the counts for superHORs are substantially lower than for HORs, we adjust the defaults for *MinCount,* and *MinWeight.*

Supplementary Note: Summary of centromeric building blocks

Monomers and *M*-blocks. CentromereArchitect infers a monomer-set *Monomers* and uses StringDecomposer to decompose a centromere into blocks with each block similar to one of monomers. Given a monomer *M* from *Monomers*, it classifies a block as an *M*-block if it is

more similar to *M* than to all other monomers. The nucleotide sequence of a monomer *M* is defined as the consensus of all *M*-blocks. MonomerGenerator attempts to cluster *M*-blocks in such a way that for any two monomers *M* and *M',* the percent identity between any two *M*-blocks is higher than the percent identity between any *M*-block and any *M'*-block. Once the monomer-set is generated, CentromereArchitect transforms the nucleotide sequence of a centromere into its monocentromere written in the alphabet of monomers.

HORs and *H*-blocks. A HOR is defined as a substring of a monocentromere. CentromereArchitect derives HORs as sufficiently frequent substrings according to a scoring function that attempts to formalize the intuition that was used for semi-manual HOR inference in previous studies. After defining HORs, CentromereArchitect rewrites the monocentromere from the alphabet of monomers into a new alphabet that combines both monomers and HORs, resulting in a HOR decomposition of centromere. For a given HOR *H,* an *H*-block is a symbol that corresponds to the *H* in the HOR decomposition of a monocentromere. Each *H*-block is also associated with a (nucleotide) fragment of the centromere.

HOR-graph and HOR-components. To allow cross-chromosomal HOR comparison, CentromereArchitect constructs a HOR-graph. Each connected component in this graph (called HOR-component) is formed by applying the single-linkage clustering to HORs that share the same monomer.

Primary and secondary HORs. For each HOR-component, CentromereArchitects selects a primary HOR and classifies all other HORs in this component as secondary HORs. It turned out that for most HOR-components, the primary HOR coincides with the canonical HOR while secondary HORs match the informal notion of structural variations of HORs (or HOR StVs, Miga et al., 2020).

Bibliography

Alexandrov, I., Kazakov, A., Tumeneva, I., Shepelev, V., & Yurov, Y. (2001). Alpha-satellite DNA of primates: Old and new families. *Chromosoma*. https://doi.org/10.1007/s004120100146

Bzikadze, A.V. and Pevzner, P.A. (2020) centroFlye: Assembling Centromeres with Long Error-Prone Reads. *Nature Biotechnology* 38, 1309-1316

Durfy SJ, Willard HF. (1989) Patterns of intra- and interarray sequence variation in alpha satellite from the human X chromosome: evidence for short-range homogenization of tandemly repeated DNA sequences. *Genomics*. 5:810-21.

Dvorkina, T., Bzikadze, A. V., Pevzner P. A. The String Decomposition Problem and its Applications to Centromere Assembly. *Bioinformatics* 2020, 36, i93–i101

Logsdon, G.A., Vollger, M.R., Hsieh, P., Mao, Y., Liskovykh, M.A., Koren, S., Nurk, S., Mercuri, L., Dishuck, P.C., Rhie, A., de Lima, L.G., Porubsky, D., Bzikadze, A.V., Kremitzki, M., Graves-Lindsay, T.A., C. Jain, Hoekzema, K., Murali, S.C., Munson, K.M., Baker, C., Sorensen, M., Lewis, A.M., Surti, U., Gerton, J.L., Larionov, V., Ventura, M., Miga, K.H., Phillippy, A.M., Eichler, E.E. (2020) The structure, function, and evolution of a complete human chromosome 8. *biorxiv,* doi: https://doi.org/10.1101/2020.09.08.285395

McNulty, S.M., Sullivan, B.A. (2018) Alpha satellite DNA biology: finding function in the recesses of the genome *Chromosome Res* 26:115–138

Miga, K.H. (2020) Centromere studies in the era of "telomere-to-telomere" genomics. *Exp Cell Res.* 394(2):112127.

Miga, K. H., Koren, S., Rhie, A., Vollger, M. R., Gershman, A., Bzikadze, A., Brooks, S., Howe, E., Porubsky, D., Logsdon, G. A., Schneider, V. A., Potapova, T., Wood, J., Chow, W., Armstrong, J., Fredrickson, J., Pak, E., Tigyi, K., Kremitzki, M., Markovic, C., Maduro, V., Dutra, A., Bouffard, G. G., Chang, A. M., Hansen, N. F., Thibaud-Nissen, F., Schmitt, A. D., Belton, J.-M., Selvaraj, S., Dennis, M. Y., Soto, D. C., Sahasrabudhe, R., Kaya, G., Quick, J., Loman, N. J., Holmes, N., Loose, M., Surti, U., Risques, R. a., Graves Lindsay, T. A., Fulton, R., Hall, I., Paten, B., Howe, K., Timp,

W., Young, A., Mullikin, J. C., Pevzner, P. A., Gerton, J. L., Sullivan, B. A., Eichler, E. E., and Phillippy, A. M. (2020). Telomere-to-telomere assembly of a complete human X chromosome. *Nature*, 585, 79-84

Shepelev, V. A., Alexandrov, A. A., Yurov, Y. B., and Alexandrov, I. A. (2009). The Evolutionary Origin of Man Can Be Traced in the Layers of Defunct Ancestral Alpha Satellites Flanking the Active Centromeres of Human Chromosomes. *PLoS Genetics*, 5(9). e1000641.

Shepelev, V.A, Uralsky, L.I, Alexandrov, A.A, Yurov, Y.B, Rogaev, E.I, Alexandrov, I.A. (2015) Annotation of suprachromosomal families reveals uncommon types of alpha satellite organization in pericentromeric regions of hg38 human genome assembly. *Genome Data* 5:139–146

Uralsky, L., Shepelev, V., Alexandrov, A., Yurov, Y., Rogaev, E., and Alexandrov, I. (2019). Classification and monomer-by-monomer annotation dataset of suprachromosomal family 1 alpha satellite higher-order repeats in hg38 human genome assembly. *Data in Brief* , 24, 103708.
